# Brachyury co-operates with polycomb protein RYBP to regulate gastrulation and axial elongation *in vitro*

**DOI:** 10.3389/fcell.2024.1498346

**Published:** 2024-11-29

**Authors:** Lilla Kokity, Zsolt Czimmerer, Bernadett Benyhe-Kis, Anna Poscher, Emese Belai, Gábor Steinbach, Zoltan Lipinszki, Melinda Katalin Pirity

**Affiliations:** ^1^ Biological Research Centre, Institute of Genetics, Hungarian Research Network, Szeged, Hungary; ^2^ Faculty of Science and Informatics, Doctoral School in Biology, University of Szeged, Szeged, Hungary; ^3^ Faculty of Science and Informatics, University of Szeged, Szeged, Hungary; ^4^ Cellular Imaging Laboratory, Core Facility, Biological Research Centre, Hungarian Research Network, Szeged, Hungary; ^5^ Synthetic and Systems Biology Unit, Institute of Biochemistry, Biological Research Centre, Hungarian Research Network, Szeged, Hungary; ^6^ National Laboratory for Biotechnology, Institute of Genetics, Biological Research Centre, Hungarian Research Network, Szeged, Hungary

**Keywords:** RYBP, mesoderm, gastruloid, polycomb complex, axial elongation

## Abstract

Early embryonic development is a complex process where undifferentiated cells lose their pluripotency and start to gastrulate. During gastrulation, three germ layers form, giving rise to different cell lineages and organs. This process is regulated by transcription factors and epigenetic regulators, including non-canonical polycomb repressive complex 1s (ncPRC1s). Previously, we reported that ncPRC1-member RYBP (RING1 and YY1 binding protein) is crucial for embryonic implantation and cardiac lineage commitment in mice. However, the role of RYBP in gastrulation and mesoderm formation has not yet been defined. In this study, we used 2D and 3D *in vitro* model systems, to analyze the role of RYBP in mesoderm formation. First, we showed that cardiac and endothelial progenitors–both derived from mesoderm–are underrepresented in the *Rybp* ^*−/−*^ cardiac colonies. In the absence of RYBP, the formation of major germ layers was also disrupted, and the expression of mesoderm- (*Brachyury, Eomes,* and *Gsc*) and endoderm-specific (*Sox17*, *Gata4*) genes was significantly downregulated. Using 3D embryoid bodies as gastrulation models, we showed that RYBP can co-localize with mesoderm lineage marker protein BRACHYURY and endoderm marker protein GATA4 and both proteins. In mutants, both proteins were detected at low levels and showed altered distribution. Additionally, we compared our *in vitro* results to available *in vivo* single-cell transcriptomes and showed that *Rybp* and *Brachyury* co-expressed in the primitive streak and six mesodermal clusters. Since caudal mesoderm exhibited one of the strongest co-expressions, we tested axial elongation in *wt* and *Rybp* ^*−/−*^ gastruloids. In the absence of RYBP, gastruloids exhibited shortened tails and low BRACHYURY levels in the tailbud. Finally, we identified BRACHYURY as a novel binding partner of RYBP and presented evidence of possible cooperative function during mesoderm formation and axial elongation. Together, our results demonstrate the previously unknown role of RYBP in mesoderm formation. We believe our findings will contribute to better understanding of the highly conserved process of gastrulation.

## 1 Introduction

Embryonic lineage commitment is a tightly regulated complex process when cells must exit pluripotency and start to differentiate. One of the most important events in early development is gastrulation, where the epiblast cells in the blastula segregate and rearrange themselves spatially to form the three major germ layers the ecto-, endo- and mesoderm. This process also involves rapid proliferation, cell migration, and the establishment of the body axes (Tam and Behringer, 1997; Bardot and Hadjantonakis, 2020). After sufficient gastrulation, the dorsal part of the embryo starts to elongate, the generation of the somites begins, and the major organs start to develop, including the brain and the heart. Due to the complexity of this process, it needs to be strictly coordinated to ensure the correct generation of the future body plan. Any errors during early development can have serious consequences in later tissue and organ development and are often incompatible with life. Multimeric protein complexes consisting of transcription factors guide the fate of cells as they progress from pluripotent state to terminally differentiated tissue types during lineage commitment. Non-canonical polycomb repressive complex 1s (ncPRC1s) are one of the key epigenetic regulators of this process (ncPRC1s). NcPRC1s can alter the epigenetic state of chromatin structure through the deposition of monoubiquitylation marks (H2AK119ub1) to compact chromatin and repress genes throughout embryonic development (Tavares et al., 2012; Wu et al., 2013). In this study, we investigated the role of RYBP (Ring1 and YY1 binding protein), also known as DEDAF (Garcia et al., 1999; Zheng et al., 2001), one of the core subunits of the ncPRC1 complexes (Gao et al., 2012) in mesoderm formation. RYBP, as a part of ncPRC1s, was shown to play an important function in multiple biological processes, including pluripotency, differentiation, and embryonic development (Pirity et al., 2005; Morey et al., 2013; Simoes et al., 2018). RYBP can also act independently from polycombs and interact with various partners to repress or activate target gene expression (Garcia et al., 1999; Schlisio et al., 2002; Li et al., 2017).

Previously, we have demonstrated that in the lack of RYBP mouse embryonic stem cells (mES cells) were unable to form contractile cardiomyocytes upon *in vitro* cardiac differentiation (Ujhelly et al., 2015), and the assembly of sarcomeric thin and thick filament proteins was disrupted (Henry et al., 2020). We have also demonstrated that the loss of cardiac transcription factor *Plagl1* could have at least partially contributed to the uncontracting phenotype. In wild type (*wt*) cells, RYBP cooperated with cardiac progenitor marker gene NKX2-5 to transcriptionally activate the P1 and P3 promoters of the *Plagl1* gene, and this activation was ncPRC1 independent (Henry et al., 2023). However, this interaction with NKX2-5 could not fully explain the phenotype, since the mRNA level of genes expressed earlier than NKX2-5 protein could be detected, was also attenuated. Results derived from the late cardiac progenitor stage (day 7) indicated low cardiac progenitor gene expression in *Rybp* null mutant (*Rybp* ^*−/−*^ *)* cardiac colonies. These all together indicated that *Rybp* might have a not yet characterized regulatory function during the early stage of cardiac lineage commitment. *Rybp* is also broadly expressed during early embryogenesis, and *Rybp* ^*−/−*^ mouse embryos die before implantation (Pirity et al., 2005), suggesting further that RYBP most likely exerts its gene regulatory effect in the progenitor stage or even earlier during gastrulation. However, the molecular mechanisms by which RYBP functions during the early stages have yet to be defined.

In this study, we used mouse ES cell and gastruloid based model systems to explore further the function of *Rybp* and characterize early cardiac progenitor and germ layer formation. First, we examined the gene expression changes using *wt* and *Rybp* ^*−/−*^ cardiac differentiated samples and showed that cardiac (*Mesp1*, *Isl-1*, *Hand1,* and *Nkx2.5)* and endothelial (*Flk-1*, *Pecam1,* and *Vcam1)* progenitor marker genes were downregulated in the *Rybp* ^*−/−*^ cardiac colonies. Next, we investigated the major germ layer marker genes and found that the mRNA levels of mesoderm (*Brachyury, Eomes,* and *Gsc*) and endoderm (*Sox17*, *Gata4*) genes were drastically decreased in the absence of *Rybp*. Using three-dimensional (3D) embryoid bodies, we detected low protein levels and altered spatiotemporal distribution of the mesoderm protein BRACHYURY and endoderm protein GATA4 in the absence of *Rybp*. To further analyze the relation between RYBP and BRCHYURY, we generated *wt* and *Rybp* ^*−/−*^ gastruloids and tested axial elongation in mutants, which exhibited a shortened tail and low BRACHYURY level in the tailbud. Finally, we identified the mesoderm protein BRACHYURY as a novel binding partner of RYBP and presented evidence of their possible cooperative function during mesoderm formation and axial elongation.

## 2 Results

### 2.1 Cardiac progenitor and cardiac endothelial lineage markers show reduced gene expression from the early progenitor stage in the lack of *Rybp*

**FIGURE 1:**
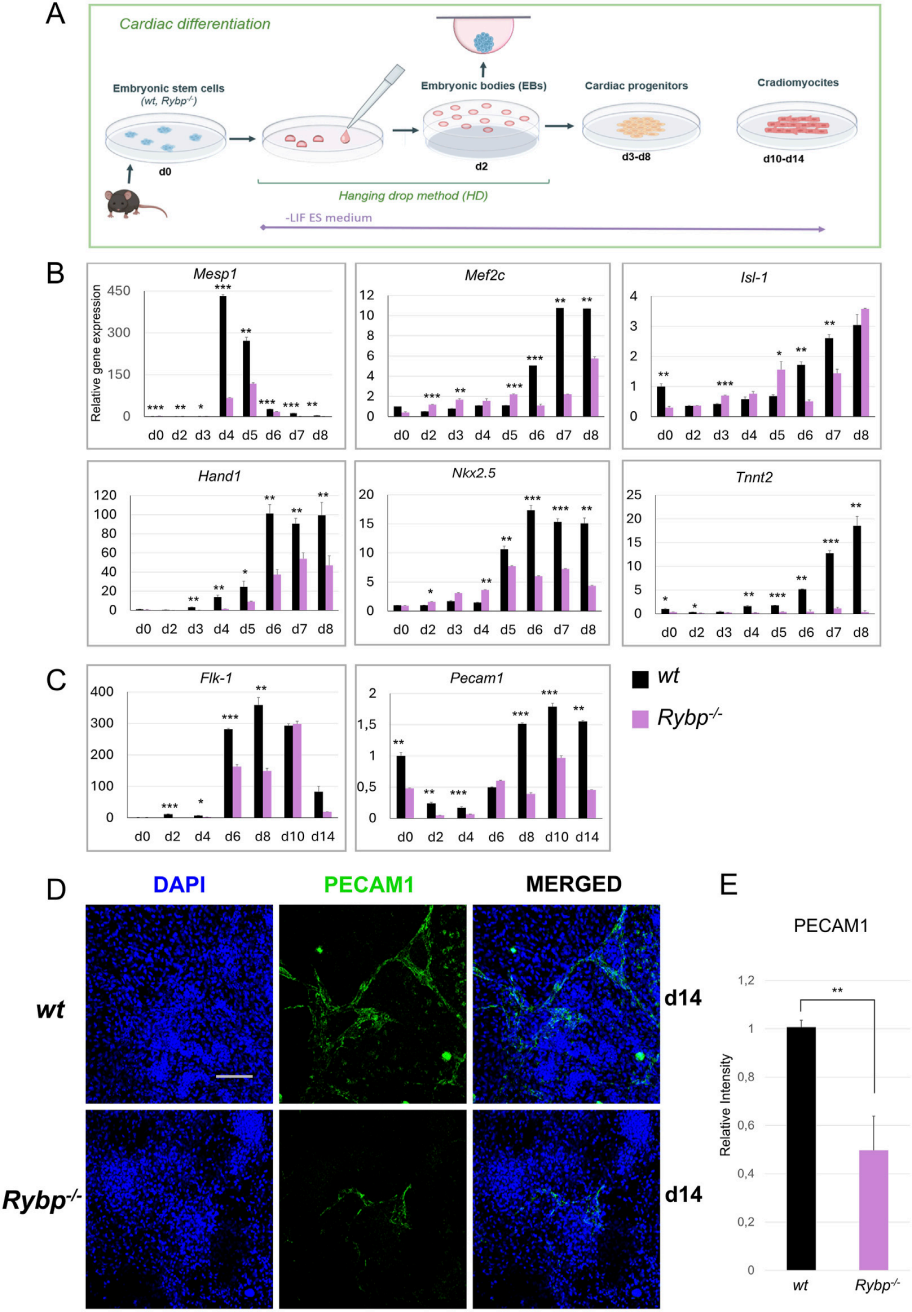
Cardiac and endothelial lineage commitment is disrupted in *Rybp* ^*−/−*^ cardiac colonies. **(A)** Schematic representation of *in vitro* cardiac differentiation. Created in BioRender. Kokity, L. (2024) https://BioRender.com/t90n837. **(B)** qRT-PCR analysis revealed a decreased expression of *Mesp1, Mef2c, Isl-1, Hand1, Nkx2.5* and *Tnnt2* cardiac progenitor and **(C)** *Flk-1* and *Pecam1* endothelial progenitor marker genes in *Rybp* ^*−/−*^ CMCs. The Ct values for each gene were normalized to the expression level of *Hprt* (Hypoxanthine phosphoribosyl transferase I) and compared to the *wt* d0 ES cells. Error bars represent standard deviation, n = 3, Values of *p* < 0.05 were accepted as significant (*p < 0.05; **p < 0.01; ***p < 0.001), Statistical method: *t*-test type 3. **(D)** Immunocytochemical analysis of PECAM1 in *wt* and *Rybp* ^*−/−*^ showed decreased PECAM1 protein level in d14 *Rybp* ^*−/−*^ CMCs. Blue: DAPI (nuclei), green: PECAM1, Olympus Confocal IX 81, Obj. 20 x; Scale bar: 100 µm. **(E)** PECAM1 immunocytochemistry signal intensities were counted from three independent samples using ImageJ software. The intensity values were normalized to DAPI signal and compared to *wt* ES cells.

During cardiac progenitor formation, early cardiac transcription factors need to be present at the right time and the right dose, therefore they often exhibit dynamic gene expression changes with tight expression windows. To comprehensively investigate the role of RYBP in progenitor formation and to visualize this rapid change, we performed *in vitro* cardiac differentiation and analyzed the gene expression of the wild *wt* and *Rybp* ^*−/−*^ colonies (Supplementary Figure S1A) on a day-to-day basis during progenitor formation. For *in vitro* cardiac differentiations, we applied the hanging drop (HD) method (Figure 1A) which doesn’t involve any inducing factors that can influence cardiac lineage commitment. It allows the generation of a variety of progenitor and mature cell types of the heart including cardiac endothelial cells and cardiomyocytes (CMCs). We checked the expression of key cardiac marker genes from day 2, when the differentiation started, till day 8, which represented the late stage of progenitor formation (materials and methods).

First, we analyzed the gene expression kinetics of Mesoderm posterior 1 (*Mesp1*), which was previously described as the earliest cardiac transcription factor and as an essential gene for cardiac mesoderm formation (Saga et al., 1996; Saga et al., 2000). In *wt* cardiac cultures rapid increase in *Mesp1* expression is expected as early as day 4 or day 5, and need to be downregulated later on (d7, d8). As expected, the *wt* cultures *Mesp1* expression was increased by day 4, sustained this high expression level only for a short period till day 5, and decayed before the late progenitor stage (Figure 1B). In the *Rybp* ^*−/−*^ cardiac colonies, *Mesp1* expression showed a significant decrease and exhibited slightly delayed kinetics compared to the *wt*. Next, we checked the gene expression of cardiac progenitor marker genes, which expression was expected to gradually increase throughout cardiac differentiation. Our result showed that in *wt* colonies, the mRNA level of late cardiac progenitor genes such as Myocyte-specific enhancer factor 2C (*Mef2c*), Heart- and neural crest derivatives-expressed protein 1 (*Hand1*), Insulin Gene Enhancer Protein ISL-1 (*Isl-1*) and Homeobox Protein Nkx-2.5 (*Nkx2-5*) increased throughout the differentiation. In the mutant cultures, we could see altered gene expression in the mRNA level of *Hand1* as well, which was low in all examined time points. The level of *Mef2c* and *Nkx2-5* mRNAs were similar in both genotypes at early time points, however from day 5/day 6, their expression in the *Rybp* ^*−/−*^ colonies couldn’t reach the level of the wild type. The expression level of *Isl-1* was diminished only on the 6th and 7th days of cardiac differentiation in comparison to the wild type counterparts, and in the rest of the timepoints were similar (d2, d8) or higher (d3, d4, d5, Figure 1B).

To provide additional evidence, we performed western blot analysis using whole cell lysates derived from *in vitro* cardiac differentiation of *wt* and *Rybp* ^*−/−*^ colonies and checked the protein kinetics of the early cardiac transcription factor MESP1 and the late cardiac transcription factor ISL-1. MESP1 protein level was examined in early progenitor stage (d2, d3, d4, d5, d6, d7) while ISL-1 protein level was analyzed in early (d2, d3, d4, d5, d6) and late time points (d7, d8, d14) of *in vitro* cardiac differentiation. Both proteins showed decreased levels and delayed kinetics in the absence of RYBP, which was consistent with the mRNA expression levels (Supplementary Figures S1B–E).

Besides cardiomyocytes, the cardiac endothelial cells also play an important role in heart morphogenesis and directly affect the performance of the adjacent cardiomyocytes (Brutsaert, 2003). This led us to question whether the endothelial lineage commitment is also compromised in the mutant. Therefore, we monitored the expression of endothelial lineage marker genes at early (d2, d4, d6) and late (d8, d10, d14) time points of cardiac differentiation. Our results showed that the expression of early endothelial receptor tyrosine kinase *Flk-1* (*Kdr, Vegfr*), which is essential for the differentiation of the endothelial cells, was decreased in *Rybp* ^*−/−*^ cardiac colonies during progenitor formation compared to the *wt* (Figure 1C). The expression of late endothelial markers such as Platelet endothelial cell adhesion molecule 1 (*Pecam1*, *CD31*) was also highly downregulated in the mutant cardiac cultures (Figure 1C). In addition, mutant samples exhibited reduced levels of PECAM1 positive cells in immunostained cardiac colonies (Figures 1D, E).

Taken together our data showed that the mRNA levels of key cardiac progenitor markers genes were significantly reduced in the lack of *Rybp.* Our results also showed that besides cardiomyocytes, the differentiation of cardiac endothelium, another mesoderm derived lineage, is also affected in the *Rybp* ^*−/−*^ cardiac colonies. In addition, the mRNA level of the earliest cardiac transcription factor *Mesp1* and the early endothelial marker gene *Kdr* was highly decreased, whose expression was expected immediately after the onset of the expression of the earliest pan-mesoderm gene *Brachyury.* These all suggested that RYBP may have a role prior to progenitor formation.

### 2.2 Lack of *Rybp* interferes with major germ layer formation during *in vitro* cardiac differentiation

**FIGURE 2:**
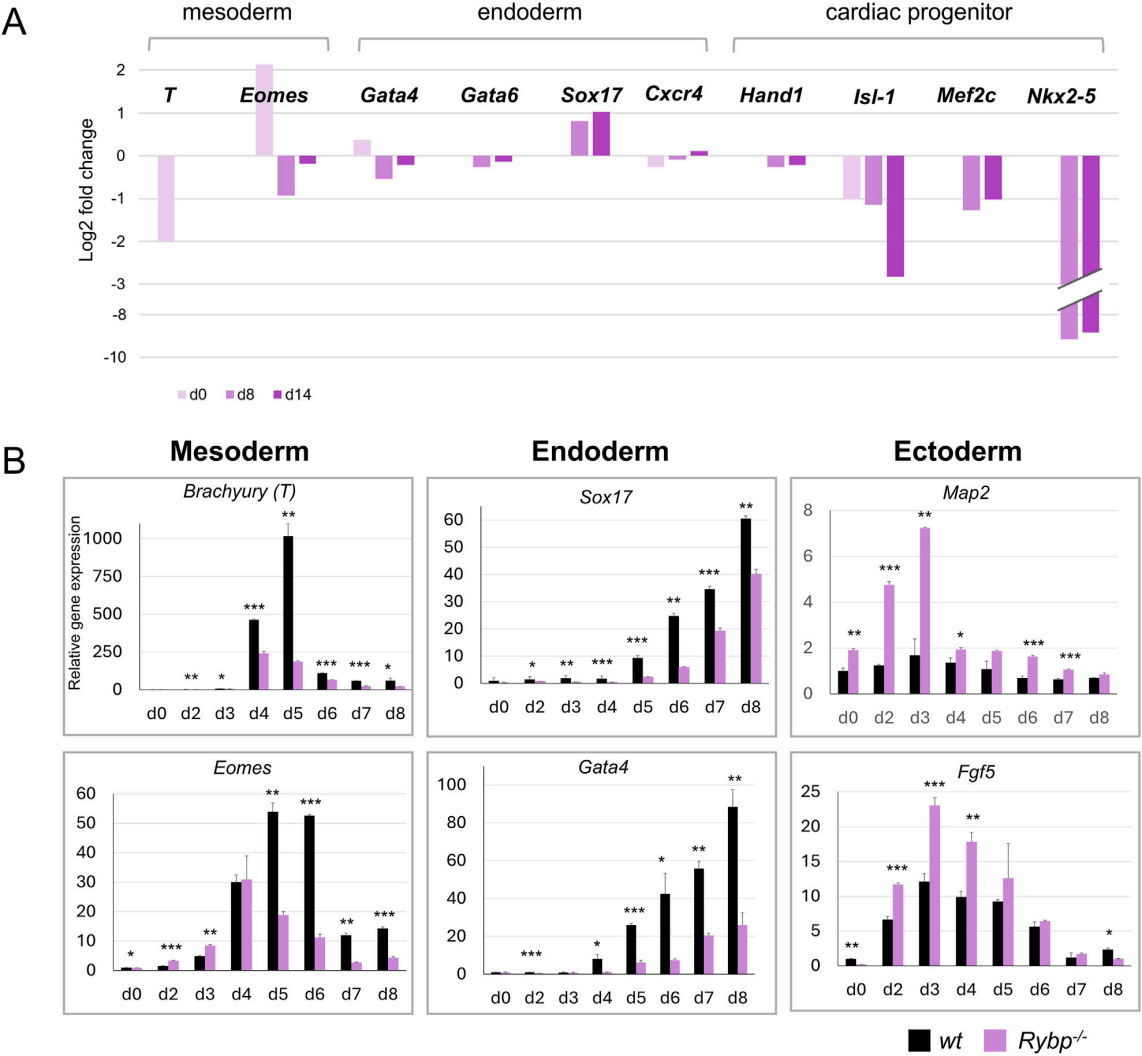
Lack of RYBP compromises the expression of major germ layers during *in vitro* cardiac differentiation. **(A)** Transcriptional changes of germ layer marker genes in d0, d8, and d14 *Rybp* ^*−/−*^ CMCs. Total transcriptome data was previously published in Ujhelly et al. (2015) and reanalyzed. The log 2 fold changes of the expression ratios of the *Rybp* ^*−/−*^ and the wild type samples were calculated and presented. **(B)** qRT-PCR analyses revealed a decreased expression of mesoderm (*Brachyury, Eomes*) and endoderm (*Sox17, Gata4*) genes and increased expression of ectoderm (*Map2, Fgf5*) genes in the *Rybp* ^*−/−*^ cardiac cultures. The Ct values for each gene were normalized to the expression level of *Hprt* (Hypoxanthine phosphoribosyl transferase I) and compared to *wt* d0 ES cells. Error bars represent standard deviation, n = 3, Values of *p* < 0.05 were accepted as significant (*p < 0.05; **p < 0.01; ***p < 0.001), Statistical method: *t*-test type 3.

To investigate if RYBP can have a potential role in mesoderm formation, first, we compared wild type and *Rybp* ^*−/−*^ whole-genome transcriptome derived from stem cells (d0), late cardiac progenitors (d8) and matured cardiomyocytes (d14) (Ujhelly et al., 2015). Although, in the lack of RYBP, transcriptomic data showed a slight reduction of mesoderm- (*Brachyury, Eomes*) and endoderm-specific (*Gata4, Gata6, Cxcr4*) gene expression, the observed changes in the late progenitor stage were not statistically significant (Figure 2A). In *wt* cultures during progenitor formation the expression of all three major germ layer marks gene are expected to increase and eventually decrease in later stages. To further analyze the mesoderm formation and confirm the perceived reduction in *Rybp* mutants, we performed qRT-PCRs every day during the time course of early (d2, d3, d4, d5, d6) and late progenitor formation (d7, d8). First, we analyzed endogenous *Rybp* expression in daily resolution and showed that *Rybp* was consistent throughout the differentiation and significant fluctuations could not been detected (Supplementary Figure S2A). However, when we compared the mesoderm marker gene expression in *wt* and *Rybp* ^*−/−*^ colonies, the T-box transcription factor *Brachyury* was found to be downregulated in the *Rybp* ^*−/−*^ cells in all examined time points (Figure 2B). *Eomesodermin* (*Eomes, Tbr2*), another T-box factor, which is also expressed in mesoderm and shares partially redundant functions with *Brachyury* was reduced from day 5 of cardiac differentiation. Besides T-box proteins, the anterior mesoderm gene *Goosecoid* (*Gsc*) was also significantly downregulated in the *Rybp* ^*−/−*^ cultures (Supplementary Figure S2B), suggesting that *Rybp* ^*−/−*^ colonies fail to form sufficient amount of mesoderm.

Similarly to the mesoderm markers, the mRNA level of endoderm markers genes such as SRY-Box Transcription Factor 17 (*Sox17)* and GATA Binding Protein 4 (*Gata4)* were also underrepresented in the *Rybp* ^*−/−*^ cultures (Figure 2B). In contrast, we found increased expression of the ectoderm marker genes such as Fibroblast Growth Factor 5 (*Fgf5*) and Microtubule Associated Protein 2 *(Map2)* in the mutant cardiac cultures (Figure 2B), which suggested that, unlike mesoderm and endoderm, the ectoderm gene expression was permitted in the *Rybp* ^*−/−*^ colonies.

**FIGURE 3:**
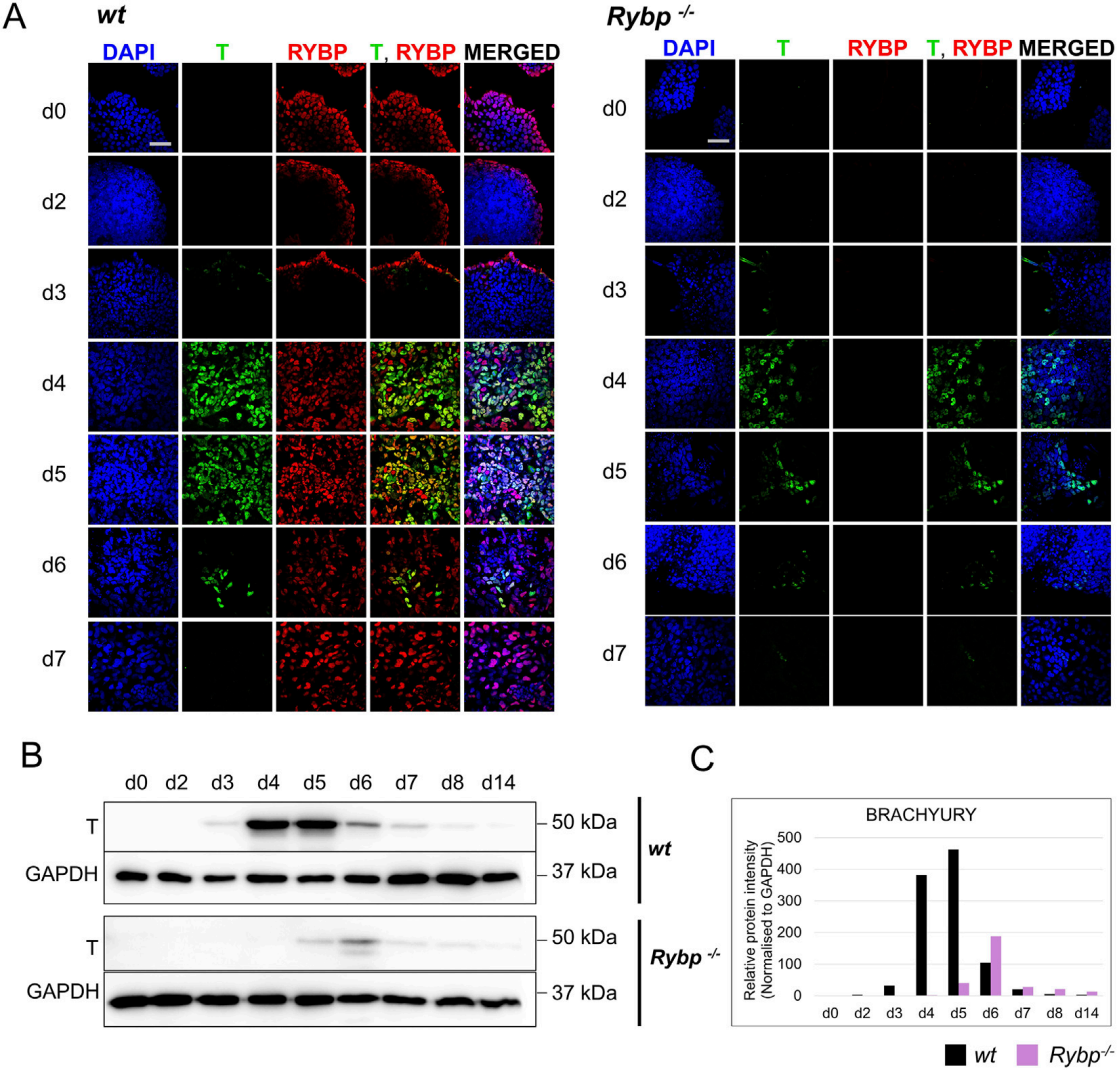
The level of BRACHYURY protein was decreased in *Rybp* ^*−/−*^ samples during *in vitro* cardiac differentiation. **(A)** Immunocytochemical analysis showed a reduced level of mesoderm marker BRACHYURY (T) in the *Rybp* ^*−/−*^ cells derived from early time points (d0, d2, d3, d4, d5, d6 and d7) of *in vitro* cardiac differentiation. Blue: DAPI (nuclei), green: T, red: RYBP. Olympus Confocal IX 81, Obj. 60 x; Scale bar: 50 µm. **(B)** Western blot analysis showed decreased T protein level in Rybp^−/−^ differentiating cardiac cultures. GAPDH was used as an internal loading control. **(C)** ImageJ quantification of western blot bands. Intensity values were normalized to GAPDH. Error bars represent standard deviation, n = 3, Values of *p* < 0.05 were accepted as significant (*p < 0.05; **p < 0.01; ***p < 0.001), Statistical method: *t*-test type 3.

After revealing the expression changes, we analyzed the distribution and subcellular localization of the mesoderm protein BRACHYURY in comparison to RYBP. In both cell lines BRACHYURY positive (+) cells were first detected at d3 in the edge of the early cardiac colonies. After d3, there was a rapid increase in the number of BRACHYURY + cells, peaking at d4 and starting to decrease after d5. In the *Rybp* ^*−/−*^ colonies, the level of BRACHYURY + cells was reduced throughout the differentiation compared to the *wt* (Figure 3A; Supplementary Figure S2C). These results are consistent with the previously described qRT-PCRs data and were further supported by western blot analysis (Figures 3B, C).

With regards to the subcellular localization of the proteins, we found that RYBP and BRACHYURY proteins were co-localized in the nuclei of the wild type cells at early time points (d3, d4, d5, d6), and the relative intensity of both proteins were fairly high in these cells. To quantify the number of RYBP and BRACHYURY double positive cells, when the BRCHYURY protein is present at its highest level, we performed flow cytometry using d4 cardiac differentiated samples. The analysis showed that 11.31% of the cells are RYBP+/BRACHYURY- only 4.44% of the cells are RYBP-/BRACHYURY+, and the majority, 72,27% of the cells are RYBP+/BRACHYURY+ (Supplementary Figure S2D).

Taken together, our data suggests that RYBP is required for proper formation of mesoderm and endoderm germ layers. The absence of *Rybp* will result in an unbalanced expression of the germ layer marker genes in favor of ectodermal lineages over mesoderm and endoderm in cardiac progenitors.

### 2.3 *Rybp* ^*−/−*^ 3D embryoid bodies fail to form proper endoderm and mesoderm germ layers

**FIGURE 4:**
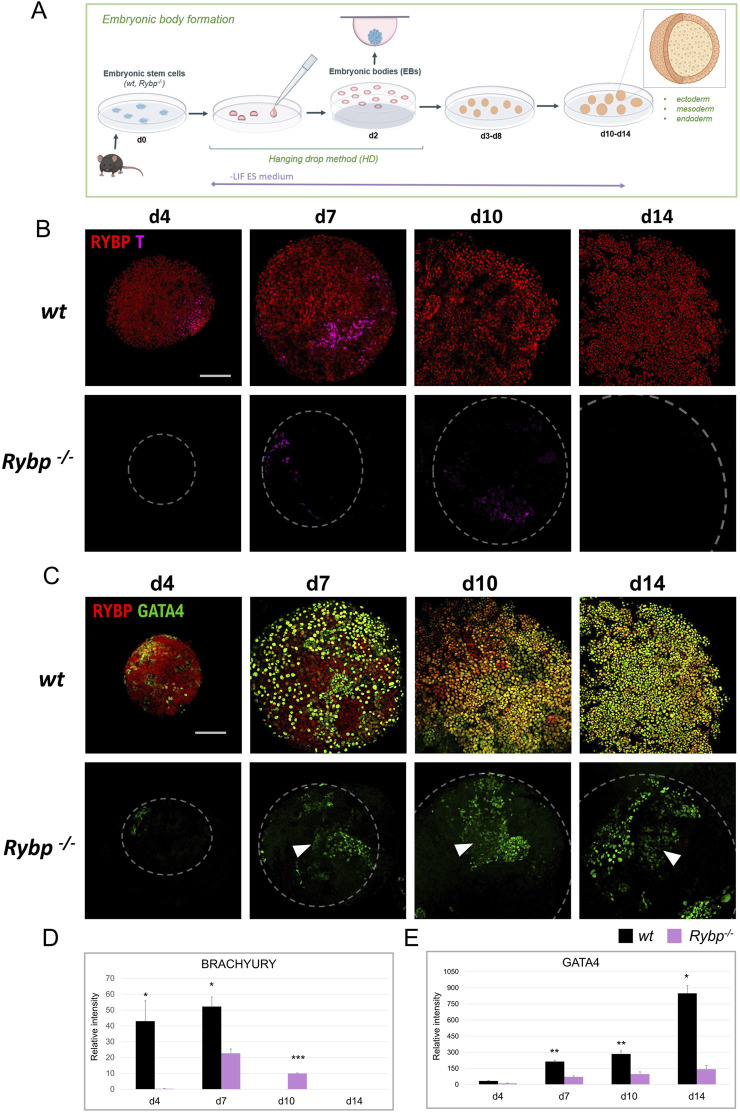
RYBP co-localizes with mesoderm marker BRACHYURY and endoderm marker GATA4 in differentiating embryoid bodies. **(A)** Schematic representation of *in vitro* embryoid body (EB) formation. Created in BioRender. Kokity, L. (2024) https://BioRender.com/b79x115. **(B)** RYBP shows nuclear co-localization with BRACHYURY and **(C)** GATA4 in 3D embryoid bodies. Whole mount immunocytochemical analysis was performed on *wt* and *Rybp* ^*−/−*^ cells derived from d0, d4, d7, d10, and d14 time points of EB formation. Blue: DAPI (nuclei), green: GATA4, red: RYBP, confocal images were taken from EBs with Olympus Confocal IX 81, Obj. 20 x; Scale bar: 100 µm. **(D)** BRACHYURY and **(B)** GATA4 immunocytochemistry signal intensities were counted from three independent EBs using ImageJ software. The intensity values were normalized to DAPI signal and compared to *wt* ES cells.

Gastrulation is a complex process which involves a series of cellular morphogenesis and cellular movements. In early embryos, the position of the cells in 3D space and the signals from the neighboring cells are also affecting the cell fate. Because of this complexity and to avoid favoring either of the germ lines, we have applied an embryoid body (EB) based 3D cell culture system and investigated the role of RYBP during the time course of EB formation (Figure 4A). EBs were generated from *wt* and *Rybp* ^*−/−*^ ES cells and spontaneously differentiated for 14 days (details in materials and methods) to form all distinctive germ layers including mesoderm and endoderm.

To reveal the spatiotemporal distribution of the mesoderm protein BRACHYURY, we performed whole mount immunocytochemistry and co-stained BRACHYURY with RYBP in d4, d7, d10, and d14 embryoid bodies (Figure 4B). In the wild type EBs, RYBP signals were detected throughout the differentiation. At d4, the majority of the cells showed medium level of RYBP signals. At d7 and d10 the intensity of RYBP signals were different as in some cells appeared higher and, in some cells, lower. At d14 most of the cells showed medium level of RYBP protein on the surface of the EBs (Figure 4B). BRACHYURY + cells were detected as early as d4 in *wt* EBs, reached the highest number at d7 (), and disappeared from d10 (Figure 4B). In contrast, *Rybp* ^*−/−*^ EBs showed decreased BRACHYURY levels throughout the differentiation. At the early progenitor stage (d4), the BRACHYURY protein was undetectable in most *Rybp* ^*−/−*^ EBs and only appeared from d7. In the mutants the BRACHYURY + cells persisted till d10 and only decayed by d14 (Figures 4B, D) in the mutants, demonstrating a delay in the BRACHYURY protein kinetics. The majority of the BRACHYURY + cells showed a nuclear co-localization with RYBP in *wt* EBs (Figure 4B; Supplementary Figures S3A, B), similarly to what we observed in two-dimensional cardiac cultures.

From d7 RYBP + cells appeared mainly in the outer layers of the EBs, where the endoderm forms, therefore we co-stained RYBP with endoderm marker GATA4 as well (Figure 4C; Supplementary Figure S4A). In wild type cells, the first GATA4+ cells were observed at d4, and the number of GATA4+ cells gradually increased during EB formation. In *wt* EBs, RYBP co-localized in nuclei with GATA4 from d4, and the co-localization sustained, resulting in a wavy multicellular outer GATA4+/RYBP + layer in later stages (Figure 4C; Supplementary Figure S4B). In the lack of RYBP there was a reduction in the number of GATA4+ cells throughout the time course of differentiation. Similarly to the *wt* EBs, GATA4+ cells started to appear at d4 in the *Rybp* ^*−/−*^ EBs, however at later time points (d7, d10, d14), mutant EBs failed to form proper GATA4+ exterior layers (Figure 4C). Besides the level of GATA4+ cells, we also observed decreased GATA4 signal intensities in the mutant EBs compared to the *wt* (Figure 4C, E white arrows).

**FIGURE 5:**
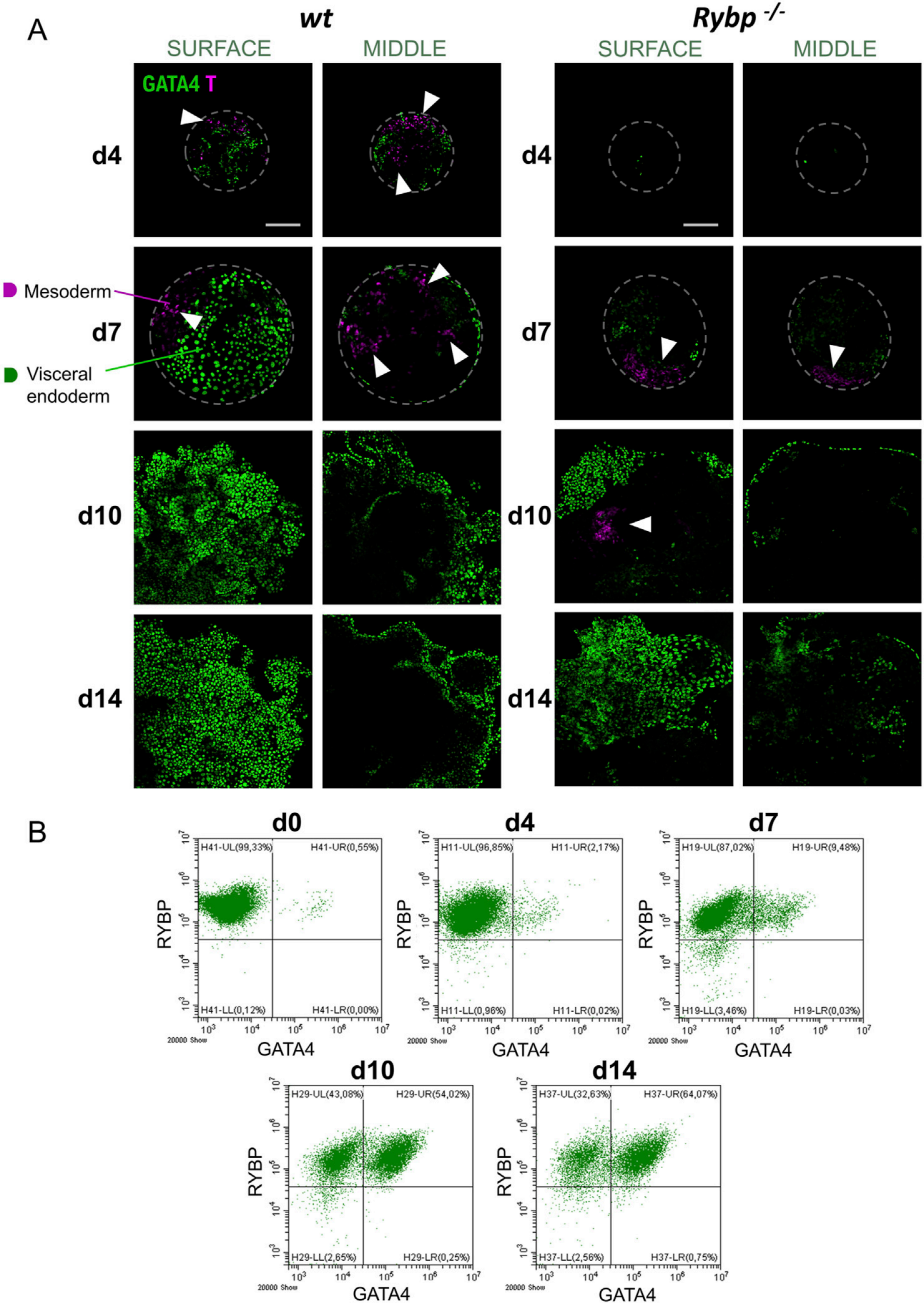
In the lack of RYBP embryoid bodies fail to form proper mesoderm and GATA4 positive visceral endoderm layer. **(A)** Immunocytochemical analysis of surface and middle EBs showed that BRACHYURY and GATA4 proteins resided in different cells during embryoid body formation and BRACHYURY + cells spread out more in *wt* EBs compared to the *Rybp* ^*−/−*^. Blue: DAPI (nuclei), green: GATA4, magenta: T, confocal images were taken from the surface and middle of the EBs with Olympus Confocal IX 81, Obj. 20 x; Scale bar: 100 µm. **(B)** Flow cytometry plots showed an increase in the number of RYBP+/GATA4+ cells over the course of EB differentiations. The percentage and standard deviation of RYBP-/GATA4-, RYBP-/GATA4+, RYBP+/GATA4-and RYBP+/GATA4+ cells. The average cell number was calculated from 3 biological samples, the representative dot plots were shown.

To gain more insights into the interplay between the two germ layers, we performed BRACHYURY and GATA4 immunocytochemistry as well and analyzed the protein localization in the surface and in the middle of the EBs. Our results showed that BRACHYURY and GATA4 proteins strictly appeared in distinct cells both in the *wt* and in the *Rybp* ^*−/−*^ EBs, and neither of the proteins showed changes in their subcellular localization in the lack of RYBP (Figure 5A). In *wt* EBs, both BRACHYURY+ and GATA4+ cells appeared on the surface and in the middle of the EBs during progenitor formation (d4, d7). In addition, within the *wt* EBs, BRACHYURY + cells spread out more compared to the mutant, where we found BRACHYURY + cells to be more restricted in one area within the EBs (Figure 5A (white arrows), Supplementary Video S1).

To quantify the number of cells co-expressing RYBP and GATA4 in *wt* EBs, we have analyzed the constituent cells of EBs from each time point (d4, d7, d10, d14) of differentiation (Figure 5B). The analysis showed that 99.33% of the cells were RYBP + at the pluripotent stage (d0). As the differentiation proceeded, a portion of the RYBP + cell population became positive to GATA4 too, and only a small portion of the cells (4.99%) lost their RYBP positivity. The number of RYBP+/GATA4+ cells increased over time and reached 64.23% of the total cell population by the 14th day of EB differentiation.

These results showed that the absence of RYBP interfered with the number of mesoderm and endoderm cells during spontaneous EB differentiation. Mesoderm and endoderm cells lacking RYBP not only presented lower levels of BRACHYURY and GATA4 proteins than the wild type but resulted in EBs with abnormal patterning as well.

### 2.4 Single-cell RNA atlas showed *Rybp* and *Brachyury* co-expression in the primitive streak, nascent mesoderm, and caudal mesoderm *in vivo*

**FIGURE 6:**
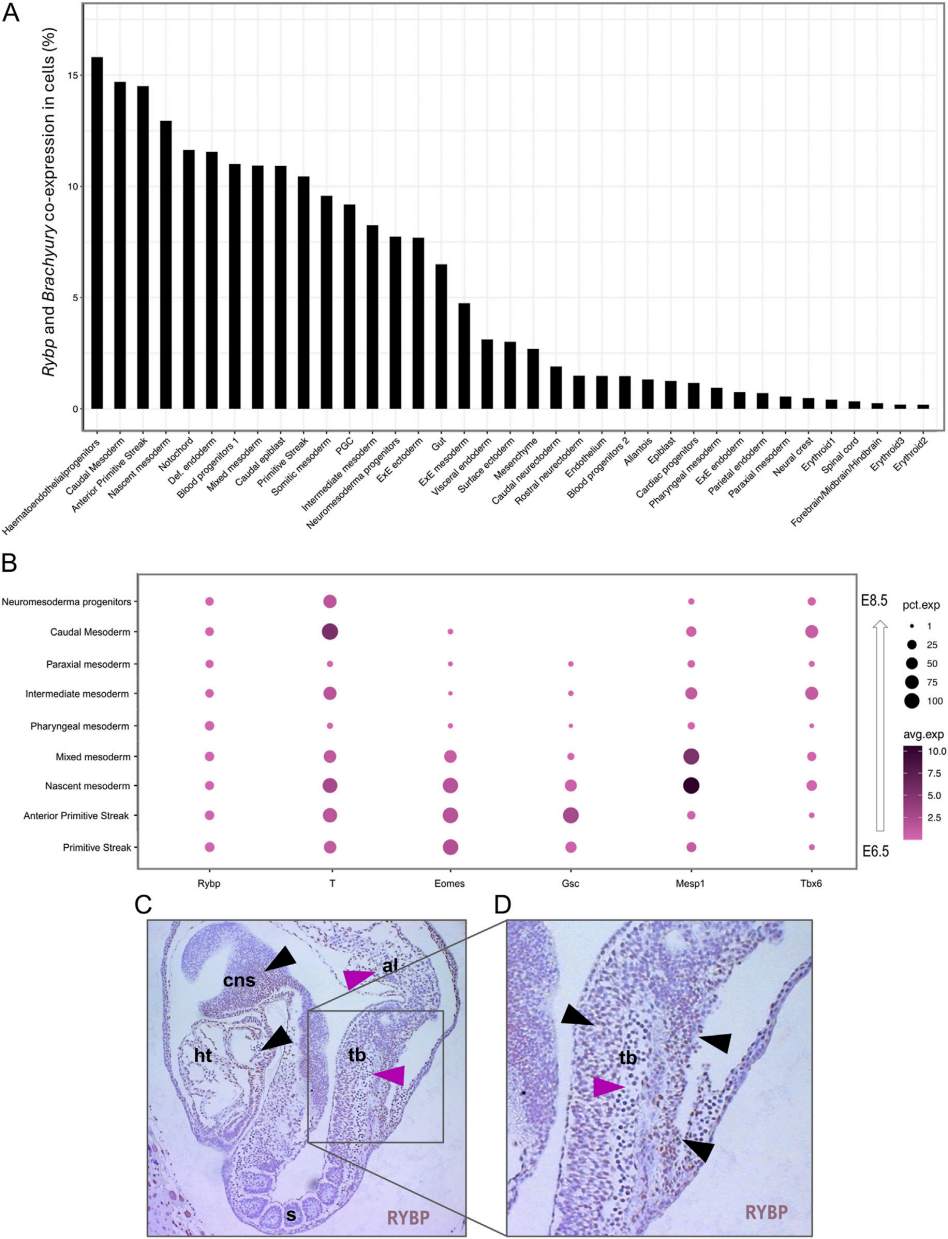
*Rybp* and *Brachyury* co-expression was detected in different mesodermal cell populations. **(A)** Bar chart showing the *Rybp* and *Brachyury* co-expression in different cell populations during mouse embryogenesis. **(B)** Dot plot of mesodermal clusters shows the expression of *Rybp, Brachyury,* and other mesodermal genes (*Eomes, Gsc, Mesp1, Tbx6*). Dot size represents the percentage of cells that express genes. Dot color shows the average level of expression. **(C)** Whole amount of immunohistochemical analysis of the E8.5 mice section showed RYBP staining in the developing central nervous system, heart, tailbud, and allantois. **(D)** RYBP + cells presented mostly in the external region (black arrows) of the mouse tailbud while RYBP- cells were detected in the internal regions (magenta arrows). Brown: RYBP. Abbreviations: cns-central nervous system; ht-heart; s-somites; tb-tailbud; al-allantois.

To compare our *in vitro* results to the *in vivo* stages, we re-analyzed a publicly available single-cell RNA sequencing (scRNA-Seq) dataset derived from wild type E6.5 – E8.5 mouse embryos (Mouse atlas was downloaded from Geo dataset: E-MTAB-6967) to gain information about cell populations expressing both *Rybp* and *Brachyury*. ScRNA-Seq datasets were processed, and cell populations were clustered as described in Pijuan-Sala et al. (2019a) (Supplementary Figure S5). The analysis revealed that *Rybp* and *Brachyury* could co-express as early as the primitive streak formation; one of the highest co-expressions was observed in the anterior primitive streak (Figure 6A), which is known to give rise to definitive endoderm and early somites (Loh et al., 2016). We also found *Rybp* and *Brachyury* co-expression in several mesodermal clusters, including the nascent, mixed, intermediate, somitic, caudal mesoderm populations and in neuromesodermal progenitors (NMP) (Martins-Costa et al., 2024). However, in other mesoderm populations, such as pharyngeal and paraxial mesoderm, there was no co-expression detected (Figure 6A). Next, we checked the overall gene expression of *Rybp, Brachyury,* and other mesoderm marker genes (*Eomes, Gsc, Mesp1, Tbx6*) in mesodermal clusters. In accordance with prior knowledge, scRNA-seq dot plots showed low, ubiquitous *Rybp* expression in all mesodermal cell populations, while the expression of *Brachyury* varied between clusters (Figure 6B). The highest *Brachyury* expression was detected in nascent mesoderm and caudal mesoderm populations. *Mesp1* and *Tbx6* were also expressed in all clusters, while other mesodermal markers such as *Eomes* and *Gsc* showed low or no expression in clusters derived from later developmental stages. These results further confirm our *in vitro* observations that *Rybp* and *Brachyury* co-express as early as the primitive streak formation, and this co-expression is sustained in nascent mesoderm, which differentiate into cardiac progenitors and eventually cardiomyocytes.

Interestingly, the caudal mesoderm cell population exhibited the second-highest co-expression in scRNA-Seq datasets. After gastrulation, the caudal mesoderm population (also called early neuromesodermal progenitors) moves posteriorly as the embryo elongates and starts to give rise to NMPs (Tzouanacou et al., 2009; Sambasivan and Steventon, 2021). It was described that both caudal progenitors and NMPs are located in the tailbud and required for proper axial elongation and somitogenesis. To gain more information on early organogenesis and check if RYBP protein is present in the tailbud, we performed RYBP immunohistochemistry (IHC) using a sagittal section of *wt* E8.5 mouse embryo. RYBP staining showed that besides the heart and brain (Figure 6C, black arrows). RYBP protein was detected in the tailbud and allantois of in E8.5 embryo (Figure 6C, pink arrows). RYBP protein was observed primarily in the external regions of the tailbud (Figure 6D, black arrows), while the cells in the interior part of the tailbud appeared to be negative (Figure 6D, pink arrow). The cells located in proximity to the allantois showed the strongest RYBP staining, and the detected signals gradually decreased in the proximal tailbud and vanished in the developing somites.

These results showed that *Rybp* and *Brachyury* co-expressed in the primitive streak and several mesoderm cell populations of *in vivo* mice. We also found that the RYBP protein is present in the tailbud, where high BRACHYURY levels are expected in developing mice.

### 2.5 *Rybp* ^*−/−*^ gastruloids fail to elongate axially, resulting in truncated tail and abnormal morphology

**FIGURE 7:**
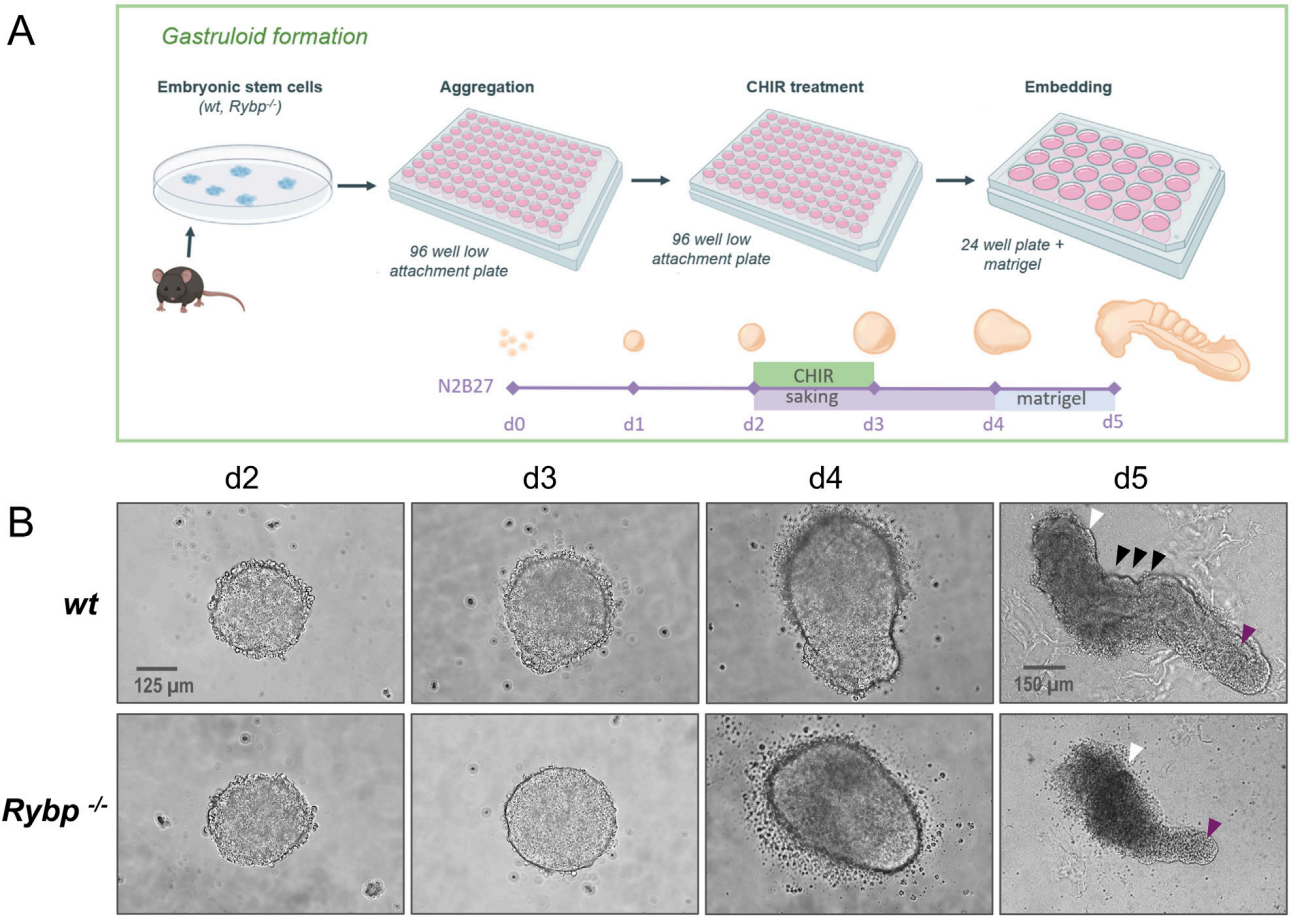
*Rybp* ^*−/−*^ gastruloids exhibited incomplete axial elongation and abnormal morphology with a truncated tail **(A)** Schematic representation of *in vitro* gastruloid formation. Created in BioRender. Kokity, L. (2024) https://BioRender.com/p16v130. **(B)** Bright field images of *wt* and *Rybp* ^*−/−*^ gastruloids show similar phenotypes during early differentiation (d2, d3, d4) and truncated tail morphology in *Rybp* ^*−/−*^ gastruloids at d5. White arrows: anterior region of gastruloids; magenta arrows: caudal region of gastruloids; black arrows: metameric structures. Spinning Disc Confocal, Obj. 20 x (d2-d4), 10x (d5), Scale bars: 125 µm (d2-d4), 150 µm (d5).

Besides its indisputable role in gastrulation and cardiac specification, BRACHYURY plays an important role in promoting segmentation and posterior axial extension during embryonic development (Wilson and Beddington, 1996). *Brachyury* heterozygote mice exhibited short-tailed phenotype (Chesley, 1935), and chimeric gastruloids generated using different percentages of *wt* and *Brachyury* null mutant cells showed reduced axial elongation (Wehmeyer et al., 2022). Considering previous results, we wanted to test whether RYBP also has a role in axial elongation. To answer this question, we generated *in vitro* gastruloids as described in van der van den Brink et al. (2020) (Figure 7A). Gastruloids were generated by aggregation of *wt* and *Rybp* ^*−/−*^ ES cells, and axial elongation was induced by 24 h CHIR99021 (CHIR) treatment to activate the Wnt signaling pathway (details in the materials and methods). We choose to use a gastruloid model system, which is capable of somitogenesis besides posterior extension and recapitulates the trunk and tail regions of E8.5 stage mouse embryos. Morphological analysis showed that *wt* and *Rybp* ^*−/−*^ gastruloids appeared indistinguishable until day 4 (Figure 7B; Supplementary Figure S6A). However, at day 5, after we embedded the gastruloids in 10% matrigel, *Rybp* ^*−/−*^ gastruloids looked less developed and failed to prolong sufficiently compared to the *wt* (Figure 7B (white arrows), Supplementary Figures S6A, S6B). The observed phenotype resembled the phenotype of the previously published *Brachyury* ^*−/−*^ gastruloids (Wehmeyer et al., 2022). The anterior region of the *Rybp* ^*−/−*^ gastruloids also differed from the *wt*, as they appeared wider, darker, and fragmented (Figure 7B, white arrows; Supplementary Figures S6A, S6B).

**FIGURE 8:**
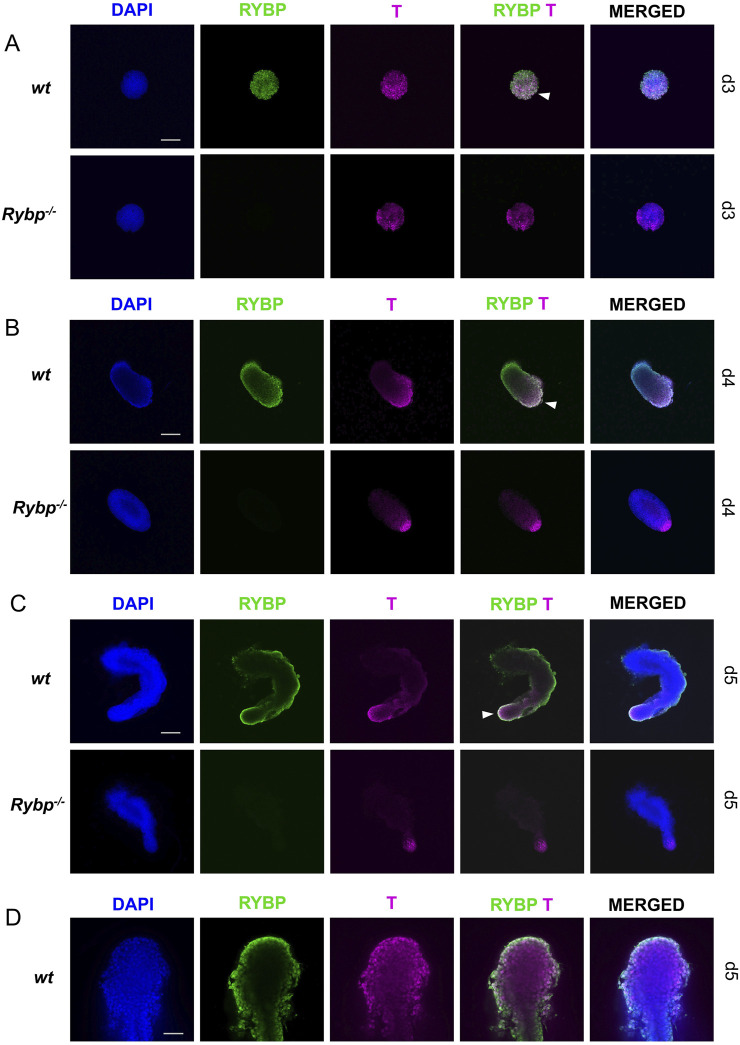
Decreased level of BRACHYURY protein was detected in the caudal region of *Rybp* ^*−/−*^ gastruloids Whole-mount RYBP and BRACHYURY immunocytochemistry of *wt* and *Rybp* ^*−/−*^ showed **(A, B)** similar BRACHYURY levels during early time points (d3, d4) and **(C)** decreased levels of BRACHYURY + cells in d5 *Rybp* ^*−/−*^ gastruloids. **(D)** RYBP and BRACHYURY proteins co-localized in the tip of the tail of d5 *wt* gastruloids. Blue: DAPI (nuclei), green: GATA4, magenta: T; Spinning Disc Confocal, Obj. 10 x **(A–C)**, 40x **(D)**; Scale bars: 200 µm **(A–C)** and 50 µm **(D)**.

Next, we performed whole-mount immunocytochemistry on the *wt* and *Rybp* mutant gastruloids to highlight the spatiotemporal distribution of BRACHYURY. In the early stages of gastruloid formation, we could not observe noticeable differences between *wt* and *Rybp* ^*−/−*^ gastruloids (Figures 8A, B). At day 3, in both cell line most of the cells were BRACHYURY+, while at day 4, the BRACHYURY + cells were oriented only to the elongating pole of *wt* and *Rybp* ^*−/−*^ gastruloids. In *wt* gastruloids at day3 and day 4 the majority of the BRACHYURY + cells were RYBP + as well. After embedding (d5), BRACHYURY positive cells were detected in the tailbud, while RYBP + cells were present throughout the exterior regions of wild type gastruloids (Figure 8C; Supplementary Figure S7B). In the mutants, we could detect fewer BRACHYURY + cells in comparison to the age-mate *wt* gastruloids. The position of the BRACHYURY + cells within *Rybp* ^*−/−*^ gastruloids did not change, they located in the caudal region of the tailbud, however the BRACHYURY + cells seemed to be restricted more to the tip of the tailbud compared to the *wt*. Using higher magnification and 3D projection, it was revealed that RYBP and BRACHYURY can co-localize in the nuclei of the *wt* cells, and the double positive cells were detected at the end of the tailbud (Figures 8C, D; Supplementary Figure S7C, Supplementary Video S2) in the same region, where caudal mesoderm cell population is expected.

In addition, we stained the gastruloids with GATA4 to monitor the endoderm layer during gastruloid formation. In early *wt* and *Rybp* ^*−/−*^ gastruloids (d3), no significant differences were observed in the amount of GATA4+ cells (Supplementary Figure S8A). At day 3, many GATA4+ cells were detected in both cell lines and the number of GATA4+ cells decreased as gastruloids started to elongate. By d4 and d5, only a few positive cells were detected in the anterior region of *wt* samples. In contrast, the number of GATA4+ cells at d4 and d5 was higher in the mutants, which suggests developmental arrest in *Rybp* ^*−/−*^ gastruloids (Supplementary Figures S8B, C and S9A, B).

**FIGURE 9:**
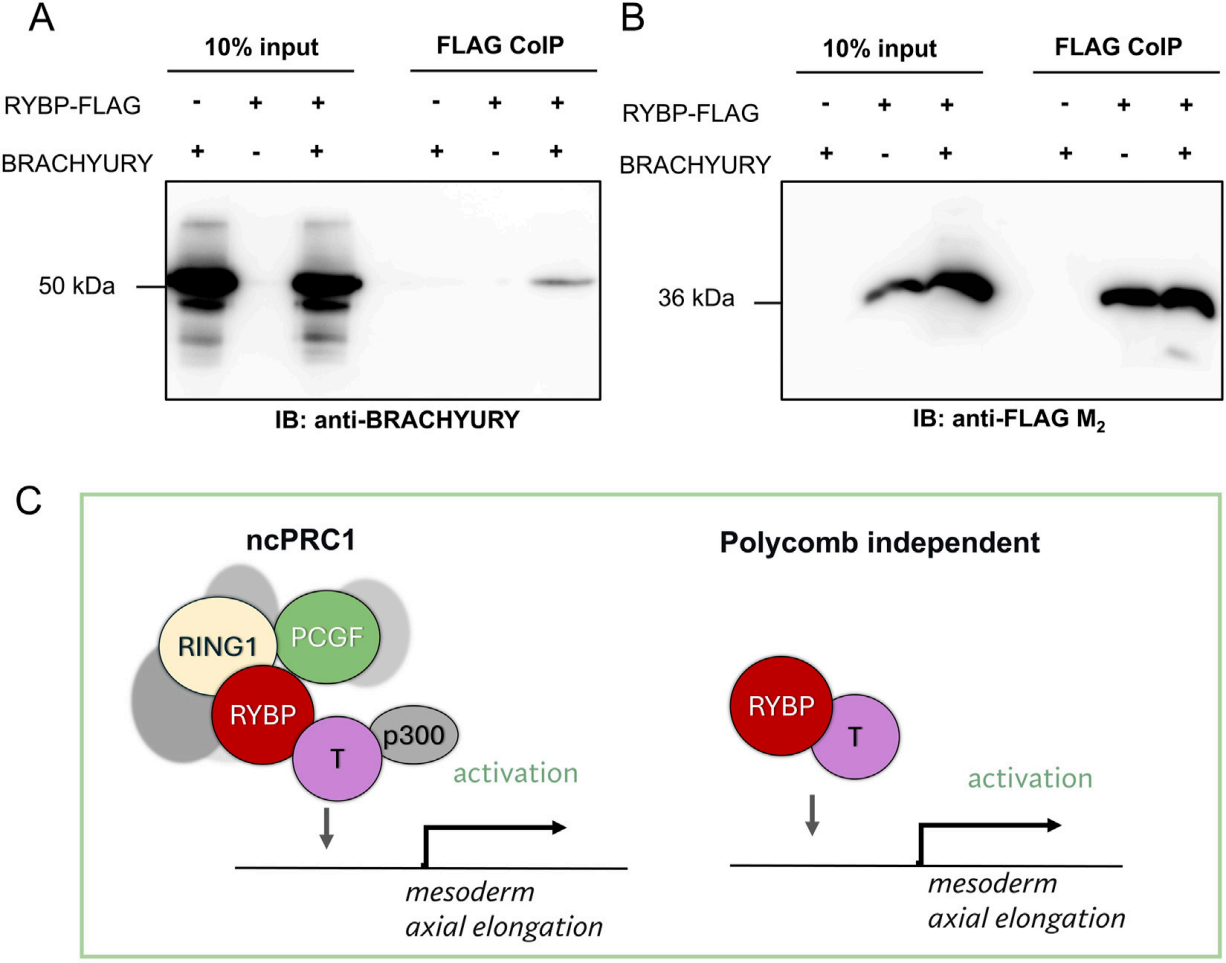
RYBP interacts with the early mesoderm protein BRACHYURY **(A)** Co-immunoprecipitation showed that RYBP and BRACHYURY physically interact with each other, which suggests that **(B)** the two proteins may co-operate in a polycomb-dependent or independent fashion to regulate mesoderm formation and axial elongation during development. **(C)** We hypothesize that RYBP and BRACHYURY may regulate mesoderm gene expression and axial elongation in polycomb dependent or independent way.

To further investigate the molecular mechanism between RYBP and BRACHYURY and to determine if RYBP can interact with BRACHYURY, we amplified and cloned the *Brachyury* cDNA to an overexpression vector (details in materials and methods) and performed co-immunoprecipitation (CoIP). CoIP results showed that RYBP physically interacts with BRACHYURY, suggesting that the two proteins may work together in a complex to regulate gene expression (Figures 9A–C).

Our results demonstrated that similarly to BRACHYURY, the presence of the RYBP protein was indispensable for proper axial elongation as well. In addition, we showed that RYBP and BRACHYURY could co-localize in the tailbud of the *wt* gastruloids. Taken together these results suggest that RYBP and BRACHYURY may share a common regulatory role in different stages of early mouse embryonic development.

## 3 Discussion

In this study, we used different *in vitro* ES cell-based model systems and provided evidence that a core ncPRC1 complex member RYBP is required during gastrulation and major germ layer formation. First, using an *in vitro* cardiac differentiation model, we revealed that the absence of *Rybp* compromises the specification of cardiac and endothelial progenitors (Figure 1). Since both progenitor cell types derive from mesoderm, this result prompted us to investigate the role of RYBP in mesoderm formation. By monitoring the relative gene expression changes, we showed that the expression of mesoderm and endoderm marker genes were severely compromised in the *Rybp* ^*−/−*^ CMCs (Figure 2). At the same time, we observed elevated ectoderm gene expression, which suggested a shift towards ectodermal lineages when RYBP was not present. A similar phenotype was described in the *Brachyury* null mutant gastrulating embryos (E7), which exhibited low mesoderm/ectoderm ratios and delayed endoderm transition compared to the *wt* littermates (Yanagisawa et al., 1981). It was shown that BRACHYURY and EOMES*,* whose expression we also found lower in our mutants, can increase the accessibility of mesoderm and endoderm programs and concomitantly suppress the neuroectoderm specification (Tosic et al., 2019). *Rypb* may also have a repressive role for ectoderm or simply a transition of meso-endo precursors into ectodermal cells. These observations suggested that decreased *Brachyury* levels could be one of the main reasons behind the observed early phenotype in the *Rybp* mutant cultures. Using the cardiac differentiation model and 3D embryoid bodies, we showed that in early differentiation stages, RYBP and BRACHYURY co-localize in the nucleus of wild type samples (Figures 2C, 3B). Immunocytochemistry of *wt* and *Rybp* ^*−/−*^ embryoid bodies also revealed that the distribution of BRACHYURY positive cells within the EBs differed in the two cell lines (Figure 5A). In the mutants, the BRACHYURY positive cells appeared to be more restricted to one region, whereas the *wt* cells spread out wider, suggesting that the absence of RYBP may also interfere with proper mesoderm cell migration. PRC1 complex members were already described to have a role in several developmental processes that require migration, e.g., PRC1.3 and PRC1.5 member AUTS2 can regulate cortical neuronal migration and neurite extension in developing brain (Gao et al., 2014; Liu et al., 2021). The role of ncPRC1 member RYBP has also been mentioned in studies related to metastatic cell migration, or tumorigenesis (Maybee et al., 2022). However, there is no available data describing the role of RYBP in migration relevant to early lineage commitment, including mesodermal cell migration. The phenotype we observed in the mutant EBs could be explained by the low expression of *Brachyury* and *Eomes* during germ layer formation (Figure 2B) since both proteins were previously described to promote mesoderm cell migration and epithelial to mesenchymal transition (EMT) (Turner et al., 2014; Arnold et al., 2008). In addition, BRACHYURY works in a dosage-dependent manner, just like RYBP, and the level of BRACHYURY directly impacts the timing of EMT and cell migration (Stott et al., 1993; Bulger et al., 2024). Besides its role in mesoderm formation, EOMES was also shown to be essential for specification to definitive endoderm (DE) during mouse gastrulation (Arnold et al., 2008), and *Brachyury* and *Eomes* double knockout (dKO) cells failed to form any type of mesoderm or definitive endoderm in *in vitro* studies (Tosic et al., 2019) similarly to what we could see in *Rybp* ^*−/−*^ (Figure 2B). The results of the present study may prompt us to investigate the role of RYBP in mesodermal cell migration in the future.

Besides BRACHYURY, our results showed fewer GATA4 positive cells in the mutants (Figures 4C, 5A). GATA4, which was previously described to mark endodermal tissues, including primitive, visceral, and definitive endoderm (Simon et al., 2018; Holtzinger et al., 2010), also co-localized with RYBP in the *wt* embryoid bodies (Figure 4C). In a similar experimental setup, it was described that GATA4 could act at a relatively early progenitor state through non-cell autonomous mechanism and significantly enhanced the generation of cardiomyocytes (Holtzinger et al., 2010; Yilbas et al., 2014). It has been described that both the mesoderm and endoderm layers were required for the proper induction of the cardiac progenitor population, therefore the decreased mRNA level of endoderm marker genes and the reduction of GATA4 protein could also be related to the impaired contractility phenotype of the *Rybp* ^*−/−*^ CMCs (Ujhelly et al., 2015; Henry et al., 2020).

In this study, we presented evidence for the first time, that RYBP was required for axial elongation of gastruloids. By generating *wt* and *Rybp* ^*−/−*^ gastruloids *in vitro*, we could bypass the early lethality of the *Rybp* ^*−/−*^ mouse embryos so that the role of RYBP in axial elongations became possible to studied. Interestingly, we observed that in the early gastrulation stage (d3, d4), the induction of the Wnt signaling pathway, which directly affects *Brachyury* expression, seems to be able to rescue the BRACHYURY level in *Rybp* mutants, however, the addition of the CHIR was not enough to restore the tailbud BRACHYURY level or to rescue the axial elongation in d5 gastruloids. *Rybp* ^*−/−*^ gastruloids exhibited shorter tails and disrupted anterior regions compared to the *wt,* and their phenotype resembled an earlier time point, which suggests a restriction in development in gastruloids in which the RYBP is not present (Figure 8). These results were also in agreement with previous studies when authors demonstrated that axial elongation required a high dosage of BRACHYURY in the tailbud of the developing mice, and a gradual decrease in the BRACHYURY level resulted in a proportionally shortened tail (Stott et al., 1993; Herrmann, 1991). During axial elongation, the BRACHYURY positive caudal mesoderm population, which is located in the tailbud, moves posteriorly as the tail grows and gives rise to neuromesodermal progenitors (van den Brink et al., 2020). This process shows a lot of similarities with gastrulation, sometimes even called “secondary gastrulation” since it requires rapid division and exit from the progenitor stage to be able to differentiate and start somitogenesis (Bardot and Hadjantonakis, 2020; Arias et al., 2022). Our result suggested that the combined presence of RYBP and BRACHYURY in the tailbud is required for the cells to be able to leave the posterior differentiation front and differentiate into somites (Figure 8).

We demonstrated that RYBP, besides regulating *Brachyury* gene expression, was also able to co-immunoprecipitate the BRACHYURY protein (Figure 9). This suggested that the two proteins might work together to regulate mesoderm development. RYBP is a multifunctional, intrinsically disordered protein (IDP), but by itself there is no proof that it can bind to the DNA (Neira et al., 2009). However, RYBP often recruits and interacts with partners, which contain specific DNA-binding domains and acts as a moonlighting protein. RYBP was shown to regulate gene expression with various partners in polycomb dependent or independent fashion. Although there are polycomb independent partners and regulations described by us (Henry et al., 2023) and others (Garcia et al., 1999; Schlisio et al., 2002; Li et al., 2017), it is more likely that RYBP and BRACHYURY function as a part of the ncPRC1s (Figure 9C). Core components of ncPRC1, including RING1B, PCGF1, PCGF2, PCGF3, and PCGF5, were described to be necessary for gastrulation and exhibited decreased *Brachyury* expression in their corresponding mutants (Voncken et al., 2003; Yan et al., 2017; Zhao et al., 2017; Morey et al., 2015; Meng et al., 2020). There are also many similarities between *Rybp* null and *Ring1b* null phenotypes during early mouse development. *Ring1b* ^*−/−*^ embryos showed mid-gastrulation lethality and null mutant embryos with inactivated cell cycle inhibitor *Cdkn2a*, which could partially rescue the *Ring1b* ^*−/−*^ phenotype, were able to grow and provide normal gastrulation however, their growth was still arrested at early somite stages, exhibiting improper axial elongation and somitogenesis similarly to what we could observe in *Rybp* ^*−/−*^ gastruloids (Voncken et al., 2003). In addition, BRACHYURY was shown to interact with p300 (Beisaw et al., 2018), which was described as a crucial member in ncPRC1-mediated gene activation, and together, they have a role in chromatin remodeling and in the activation of mesoderm specification (Koch et al., 2017).

Taken together, we combined different *in vitro* approaches to shed light on the early developmental role of core ncPRC1 member RYBP with a special focus on mesodermal lineages. We demonstrated that RYBP was required for proper germ layer formation co-localized with the earliest mesodermal protein BRACHYURY during gastrulation and axial elongation. This suggests their possible cooperative role in early development. The combination of different model systems gave us more insight into the early embryonic phenotype, which otherwise would be difficult to study, and increased our knowledge of a developmental gene with early lethality. Presumably, the presented results contribute to the understanding of the highly conserved process of gastrulation and axial elongation and provide additional information about the activation role of Polycomb group proteins in these processes.

## 4 Materials and methods

### 4.1 Cell lines and culture condition

Mouse (129SV/Ola) R1 ES cells (mentioned as a wild type or *wt*) and R1 derived *Rybp* null mutant ES cells (mentioned as *Rybp* ^*−/−*^) were thawed on mitomycin C (Sigma, #M0503) subjected MEF feeder layer and cultured as previously described by Henry et al. (2020).

HEK293 were maintained in Dulbecco’s Modified Eagle Medium (DMEM, Gibco, # 31966047) supplemented with 10% fetal bovine serum (Pan-Biotech, # 3702), 0.1 mM MEM Nonessential Amino Acids (Gibco, #11140-068), 1% sodium pyruvate (Gibco, #11360-039) and 50 U ml−1 penicillin/streptomycin (Gibco, #15140-122). The cells passage a 70%–80%, and the medium was changed every second day.

All the cells were cultured in humidified conditions containing 5% CO_2_ at 37°C.

### 4.2 *In vitro* 2D cardiac differentiation

For *in vitro* cardiac differentiation, embryoid bodies (EBs) were generated by the hanging-drop (HD) method (Keller, 1995). The ES cells were dissociated from the monolayer using 0.05% Trypsin-EDTA (Gibco, #15400-054) and counted with a Bürker chamber. The cell suspension was diluted to 50 cells/μ l in a differentiation medium (ES medium without LIF), and 20 μ l droplets were dispensed to the lids of 10 cm bacterial dishes, where each droplet contained 1000 cells. The bacterial dishes were filled with Dulbecco’s phosphate-buffered saline (DPBS (1x), Gibco, #14190–094) to prevent the droplets from drying out. By reversing the lid of the dish, the cells were allowed to aggregate for 48 h with the help of gravity. At the second day, the EBs were collected and plated into a 6 cm 0.1% gelatine-coated cell culture dishes (SPL Life Sciences, #20060) for gene expression and protein studies and 24-well plate (Corning, #356230) containing 0.1% gelatine-coated coverslips for immunocytochemistry (ICC). The cells were maintained up to 8 or 14 days in a differentiation medium, which was changed every second day. The samples were collected at different time points of cardiac differentiation and ES cells were collected to represent the pluripotent stage.

### 4.3 *In vitro* 3D embryoid body (EB) formation

Embryoid bodies were generated by the HD method in the same regard as described above in cardiac differentiation. The EBs were collected on the second day into 10 cm diameter bacteriological dishes (SPL Life Sciences, #20100) where attachment of the cells was prevented. The EBs were kept in suspension for 14 days and the medium was changed every second day. The EBs were harvested for ICC analysis on day 4, day 7, day 10, and day 14 (labeled as d4, d7, d10, d14) during EB differentiation.

### 4.4 Quantitative real-time PCR (qRT-PCR)

Total RNA was isolated from ES cells and *in vitro* cardiac differentiated samples using the Gene Jet RNA Purification Kit (Thermo Scientific, #K0732) according to the manufacturer’s instructions. For a detailed examination of gene expression in the time course of progenitor formation, samples were collected every day from day 2 till day 8. To check the expression of endothelial marker genes, the samples were derived from day 2, day 4, day 7, day 10, and day 14 (d2, d4, d7, d10, d14), where d2, d4, and d7 represent the early stage and d10 and d14 represent the late stage of differentiation. cDNA synthesis was achieved with the isolated RNA using a High-Capacity cDNA Reverse Transcription Kit (Applied Biosystem, #4368814) according to the manufacturer’s instructions. qRT-PCR was performed in SYBR Select Master Mix for CFX (Applied Biosystems, #4472942) using the Roto-Gene Q PCR machine (QUIAGEN). Relative gene expression changes were determined using the ΔΔCt method. The Ct (cycle threshold) values for each gene were normalized to the expression level of *Hprt* (Hypoxanthine phosphoribosyl transferase I) as an internal control. To calculate the fold expression changes the values were compared to the expression of wild type d0 ES cells. The primers used in this study are listed in the Supplementary Table S1.

### 4.5 Western blot analysis

Protein samples for western blot analysis were collected from ES cells and from different time points of *in vitro* cardiac differentiation. Protein kinetics were analyzed in the time course of progenitor formation (d2-d7), and in the case of ISL-1 and BRACHYURY proteins, the time points were extended to later stages (d8, d10, d14) as well. From the designed time points total protein was extracted using RIPA-buffer supplemented with protease inhibitor (Sigma, #S8830). The concentration of the lysates was determined with Bradford assay (Bradford, 1976) and until usage the samples were stored in 6x Laemmli dye (Laemmli, 1970). Equal protein quantities (20 μg each) were loaded into a 12% sodium dodecyl sulfate-polyacrylamide, and gel electrophoresis (SDS-PAGE) was performed using Bio-Rad Mini-PROTEAN 3 Cell (#165-3301). The protein was then transferred to a polyvinylidene fluoride membrane (Immobilon-P, Merk Millipore, #IPVH00010) and blocked in 5% milk for 1 h at room temperature. Afterwards, the membranes were incubated overnight in 5% milk supplemented with primary antibodies (anti-MESP1 (Santa Cruz, #sc130461, 1:1000), anti-ISL-1 (Santa Cruz, #sc390793, 1:1000), anti-BRACHYURY (R&D Systems, #AF 2085, 1:1000), anti-FLAG M2-IgG1-HRP (Sigma, #A8592, 1:4000) antibodies and anti-GAPDH (Bio-Rad, #AHP1628T, 1:1000) was used as housekeeping protein. Goat-anti-mouse IgG-HRP conjugate (Bio Rad, #1721011, 1:2000), goat-anti-Rabbit IgG-HRP conjugate (Invitrogen, #G-21234, 1:10000) and donkey-anti-Goat IgG-HRP (Invitrogen, #A15999, 1:2000) were used as the secondary antibodies. The membranes were washed five times with TBST buffer and developed using Immobilon Western Chemiluminescent HRP Substrate (Merk Millipore, #WBKLS0500), and signals were captured with Alliance Q9 system (UviTech).

### 4.6 Immunocytochemistry

For immunofluorescence staining, *in vitro* cardiac cultures and EBs were fixed with 4% paraformaldehyde (PFA, Sigma-Aldrich, #P6148) for 20 min at 4°C. The cells were permeabilized in 0.2% Triton X-100 (Sigma-Aldrich, #T8787-250ML) in PBS for 20 min at RT. After permeabilization, the cells were blocked in 5% Bovine Serum Albumin (BSA) (VWR Life Science, #9048-46-8) in PBS for 1 h at RT. Sequentially the samples were incubated overnight in 5% BSA supplemented with the primary antibodies (anti-PECAM1 (R&D Systems, #AF3628, 1:1000), anti-BRACHYURY (R&D Systems, #AF2085, 1:1000), anti-DEDAF/RYBP (Merck Millipore, #AB3637, 1:1000), anti-GATA4 (Anti-GATA4 antibody, Thermo-Fisher, #14-9980-82, 1:1000)) in 4°C under gentle shaking. The next day the cells were washed five times with PBS, blocked in 5% BSA for 1 h and incubated in 5% BSA containing fluorescently labeled secondary antibodies (Alexa Fluor 488 Goat anti-Mouse (Invitrogen, #A10667, 1:2000) Alexa Fluor 488 Donkey anti-Rabbit (Invitrogen, #A21206, 1:2000), Alexa Fluor 647 Donkey anti-Goat (Invitrogen #A21447, 1:2000), Alexa Fluor 568 Donkey anti-Rabbit (Invitrogen, #A10042, 1:2000), and Alexa Fluor 488 Donkey anti-Rat (Invitrogen #A21208,1:2000)) for 1 h at 4°C. The cells were then incubated 20 min in PBS containing 4’6-diamidino-2-phenylindole (DAPI; Vector Laboratories, #H-1200, 1:2500) for nuclear visualization. The samples were washed three times with PBS and mounted in Fluoromount-G (Invitrogen, #00-4958-02). The images were taken in Olympus LSM confocal microscopy (Olympus Corporation, Japan).

### 4.7 ImageJ quantification of western blot and immunocytochemistry images

Westen blot and immunocytochemistry images were quantified using ImageJ software. Western blot band intensities were normalized to GAPDH internal controls, and the intensity values were compared to *wt* ES cells. All immunocytochemistry standings were analyzed in triplicates derived from three independent samples and the intensity values were normalized to corresponding DAPI counterstaining and compared to *wt* ES cells. To quantify the 3D EBs and gastruloids we measured the merged z-stack images of the samples.

### 4.8 Flow cytometry analysis (FC)

Single-cell suspension was collected from ES cells, d4 cardiac differentiated, and d4, d7, d10, and d14EBs. The samples were fixed in 70% ethanol for 20 min and washed with 0, 5% BSA. The cells were overnight stained with anti-DEDAF/RYBP (Merck Millipore, #AB3637, 1:1000) in combination with anti-BRACHYURY (R&D systems, #AF 2085, 1:1000) in cardiac samples and in combination with anti-GATA4 (Anti-GATA4 antibody, Thermo-Fisher, #14-9980-82, 1:1000) in EBs. Alexa Fluor 488 Donkey anti-Rabbit (Invitrogen, #A21206, 1:2000), Alexa Fluor 647 Donkey anti-Goat (Invitrogen #A21447, 1:2000), Alexa Fluor 647 Goat anti-Rabbit (Invitrogen, #A21244,1:2000), and Alexa Fluor 488 Donkey anti-Rat (Invitrogen #A21208,1:2000) secondary antibodies were used according to the host species. All antibodies were diluted in dilution solution [0.5% BSA and 0.5% TWEEN-20 (Sigma-Aldrich, #P9416-100ML)]. Fluorescence signals were measured of 50,000 cells/sample using Beckman Coulter CytoFLEX Flow Cytometer (Beckman Coulter, United States) with appropriate settings and compensation adjustments. The negative control was utilized to establish the negative boundary, while the fluorescence intensity and number of positive cells were measured. Subsequently, the data were analyzed using CytExpert 2.4 software.

### 4.9 Single-cell RNA-seq analysis

The single-cell RNA-seq dataset derived from the different developmental stages of mouse embryos was collected from ArrayExpress (accession number: E-MTAB-6967, file name: atlas_data.tar.gz) for our analysis. The metadata generated by the original pipeline was used for our downstream analysis (Pijuan-Sala et al., 2019b), which was performed in RStudio (v.2024.04.02.) with the Seurat package (v.5.1.0). We filtered the dataset to include only cells with clearly defined cell types, then recreated the uniform manifold approximation and projection (UMAP) plot based on the clustering information and coordinates identified by Pijuan-Sala et al. (2019a) with DimPlot function from the Seurat package (v.5.1.0). The resulting 116,312 embryonic cells were classified into 37 clusters, and further analysis was performed accordingly. The bar chart was generated using the *Rybp* and *Brachyury-*positive cells of the previously identified 37 clusters. Double positive cells were classified by normalized expression value higher than 0. The plot was performed with ggplot2 (v.3.5.1). Primitive streak and mesoderm clusters were selected for gene expression pattern analysis of marker genes. The average gene expression values were obtained using the DotPlot function from the Seurat package (v.5.1.0), and a percentage cutoff of 1 was applied; the visualization was executed using ggplot2 (v.3.5.1).

### 4.10 Histology and immunohistochemistry

Wild type E8.5 mouse embryos were fixed in 4% PFA overnight, and paraffin-embedded sections (6 μm) were mounted for staining. For immunohistochemistry, deparaffinized tissue slides were first treated with 3% H2O2 for 30 min to inactivate endogenous peroxidases, then washed with double-distilled H_2_O and soaked in PBS for 5–10 min. The slides were blocked with 10% BSA and then exposed to anti-RYBP antibody (Millipore, #AB3637, 1:100) overnight at 4°C. After the excess antibody was removed, the samples were incubated with a 1:400 dilution of biotin-conjugated secondary anti-rabbit (Vector labs) antibodies for 45 min at room temperature, washed in PBS, and incubated with avidin-biotinylated enzyme complex for 45 min. The reaction was developed with a DAB kit (Vector labs) and monitored by microscopy for the proper exposure.

### 4.11 Gastruloid formation

Gastruloids were generated as previously described in van den Brink et al. (2020). Briefly, *wt* and *Rybp* ^*−/−*^ ES cells were aggregated in ultra-low 96 well plates (Corning, #7007) containing N2B27 (Ndiff 227, #Y40002) medium. On the second day, the cells were treated with 3 μM CHIR90021 (Selleck Chemcals, #S1263) for 24 h, then the medium was changed back to N2B27. At day 4 gastruloids were collected and embedded in 10% matrigel (Corning, #356230) 90% N2B27 medium mixture in 24 well cell culture plates (Corning, #3524). The samples were fixed with 4% PFA in different time points (d2, d4, d5) during gastruloid formation and immunocytochemistry was performed as described above.

### 4.12 Cloning of prk7-Brachyury overexpression vector

Full-length of *Brachyury* cDNA was amplified from d4 *wt* cardiac differentiated cells (where the highest *Brachyury* gene expression was expected) with PrimeSTAR GXL DNA Polymerase (Takara Bio, #R050Q) using 5′-TAT​AAA​GCT​TTG​TTG​GGT​AGG​GAG​TCA​AGA-3′ forward and 5′-TTT​TAA​GCT​TAT​AGA​TGG​GGG​TGA​CAC​AGG​T-3′ reverse primers. The amplified cDNA was then cloned in the prk7 overexpression vector using HindIII restriction sites. The sequence accuracy and the orientation of the cDNA construct were confirmed by restriction digestion and sequencing.

### 4.13 Co-immunoprecipitation (CoIP)

HEK293 cells were transiently transfected with 5 μg prk7-*Brachyury* and pcDNA3.1-*Rybp*-FLAG using Calcium phosphate (CaPO4) method as described in Henry et al. (2023). The cells were harvested 2 days after transfection and were washed with PBS and lysed by 10x passing the cell suspension through a G26 needle in EB buffer (50 mM HEPES (Merck, #1.12041), 150 mM NaCl (Sigma, #S-5886), 0.5 mM MgCl_2_ (Roanal, #13007), 0.1% NP40 (Sigma-Aldrich, #74385), 5% glycerol (Sigma, #G-9012), 0.5 mM DTT (Thermo Scientific, #R0862), 1 mM protease inhibitor cocktail (Sigma, #S8830), 25 μM MG132 (Cayman, #CAYM10012628), 0.1 μl/mL benzonase nuclease (Merk-Millipore, #70746-3). Then the lysates were centrifuged (17,000 *xg*, 20 min, 4°C), and 10%f the cleared supernatant was stored as input, and the rest was used for co-immunoprecipitations (CoIP). CoIP was performed using anti-FLAG-M_2_ magnetic beads (Sigma-Aldrich, #M8823) at 4°C for 90 min. Bound proteins were eluted with 200 µg/µ l 3xFLAG peptide (Sigma-Aldrich, #F4799) through competition elution, and Laemmli sample buffer was added to the eluted proteins followed by boiling for 5 min. The samples were then loaded into a 10% SDS-PAGE, and western blot analysis were carried out as described above using the indicated antibodies. For CoIP experiments anti-BRACHYURY (R&D Systems, #AF 2085, 1:1000) and anti-FLAG M2-IgG1-HRP (Sigma, #A8592, 1:4000) antibodies were used as primary antibodies donkey-anti-Goat IgG-HRP (Invitrogen, #A15999, 1:2000) were used as the secondary antibodies.

### 4.14 Statistical analysis

All experiments were repeated three times, and technical repeats were used as triplicates at each examined timepoints. Experiments were evaluated with Microsoft Excel by using Student’s t-test type 3. Means are standard deviation. Values of *p* < 0.05 were accepted as significant (*p < 0.05; **p < 0.01; ***p < 0.001).

## Data Availability

The publicly available, published scRNA-seq data set analyzed in this study can be accessed on E-MTAB-6967 accession number.
