# Supplementary material for: Brachyury co-operates with polycomb protein RYBP to regulate gastrulation and axial elongation in vitro
Source: Front Cell Dev Biol. 2024 Nov 29;12:1498346. doi: 10.3389/fcell.2024.1498346 (PMC11638158; doi:10.3389/fcell.2024.1498346)
Supplement: Supplementary file 1 [file Table1.docx]

**Supplementary material**

| **Genes** | **Forward primers** | **Reverse primers** |
| --- | --- | --- |
| *Hprt* | 5’- AGTCCCAGCGTCGTGATTAG -3’ | 5’- GCAAGTCTTTCAGTCCTGTCC -3’ |
| *Rybp* | 5’- TTAGGAACAGCGCCGAAG -3’ | 5’- GCCACCAGCTGAGAATTGAT -3’ |
| *Mesp1* | 5’- GATCCCCGCCTGCCTA -3’ | 5’- CTGAAGAGCGGAGATGAGG -3’ |
| *Mef2c* | 5’- GGTGCTGACGGGAACAACTT -3’ | 5’- CACTTCTTCACTGCCACAGC -3’ |
| *Isl-1* | 5’- GGGATGGGAAAACCTACTGTAAAAGAGA -3’ | 5’- GTCGTTCTTGCTGAAGCCTATGCTG -3’ |
| *Hand1* | 5’- CCAAGGATGCACAAGCAGGTGAC -3’ | 5’- TGCGCCCTTTAATCCTCTTCTCG -3’ |
| *Nkx2.5* | 5’- TACACCTCTAATGTCCTCCCTTG -3’ | 5’- CCATACAGTTGACTTCCCAACAC -3’ |
| *Tnnt2* | 5’-CTGAGACAGAGGAGGCCAAC-3’ | 5’-ACCAAGTTGGGCATGAAGAG-3’ |
| *Flk-1* | 5’- TGTGTTTCCTTAGATCGCGC -3’ | 5’- CAAAGAAGTCACAGAGGCGG -3’ |
| *Pecam1* | 5’- CATGGGAGGTGATGAATGGG -3’ | 5’- TGCATTTGTACTTCCCGGAG -3’ |
| *Brachyury* | 5’- TACACCTCTAATGTCCTCCCTTG -3’ | 5’- CCATACAGTTGACTTCCCAACAC -3’ |
| *Eomes* | 5’- ACCGCCCACTACAATGTTTT -3’ | 5’- TGCCCTGCATGTTATTGTCC -3’ |
| *Gsc* | 5’- TCCAGGAGACGAAGTACCCAGACGT -3’ | 5’- CTCGGCGGTTCTTAAACCAGACCT -3’ |
| *Sox17* | 5’- GCCAAAGACGAACGCAAGCG -3’ | 5’- TTCTCTGCCAAGGTCAACGCCT -3’ |
| *Gata4* | 5’- CTCTATCACAAGATGAACGGCATCAAC -3’ | 5’- TCTGGCAGTTGGCACAGGAGAG -3’ |
| *Map2* | 5’- AAAGAGAACGGGATCAACGG -3’ | 5’- TTGTGTTGGGCTTCCTTCTC -3’ |
| *Fgf5* | 5’- CAAAGTCAATGGCTCCCACGAAG -3’ | 5’- CTACAATCCCCTGAGACACAGCAAATA -3’ |

**Supplementary table 1. Primer sequences**
