# Supplementary material for: Brachyury co-operates with polycomb protein RYBP to regulate gastrulation and axial elongation in vitro
Source: Front Cell Dev Biol. 2024 Nov 29;12:1498346. doi: 10.3389/fcell.2024.1498346 (PMC11638158; doi:10.3389/fcell.2024.1498346)
Supplement: Supplementary file 2 [file DataSheet1.pdf]

BRACHYURY co-operates with polycom protein RYBP to regulate gastrulation and axial elongation *in vitro*

Supplementary Figures

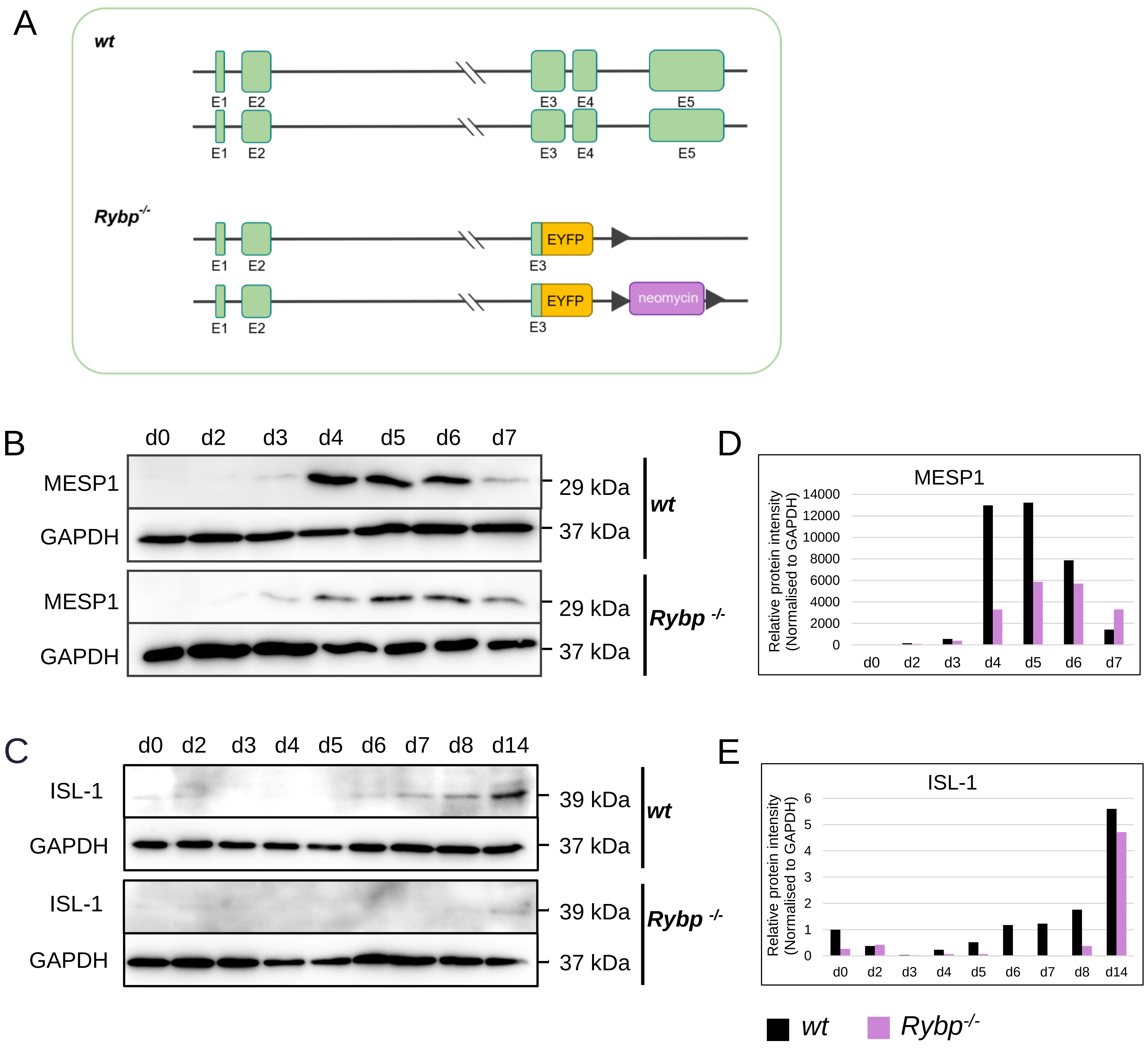

Supplementary Figure 1. *Rybp$^{-/-}$* cardiac differentiated samples exhibited decreased MESP1 and ISL-1 protein level

(A) Schematic representation of *wt* and *Rybp$^{-/-}$* ES cell lines. **(B)** Western blot analysis showed decreased MESP1 and **(C)** ISL-1 protein levels in *Rybp$^{-/-}$* differentiating cardiac cultures. In Western blots GAPDH was used as an internal control**. (D) (E)** The western blot band intensities were quantified using Image J software, and the intensity valuse were normalized to the corresponding GAPDH intensities.

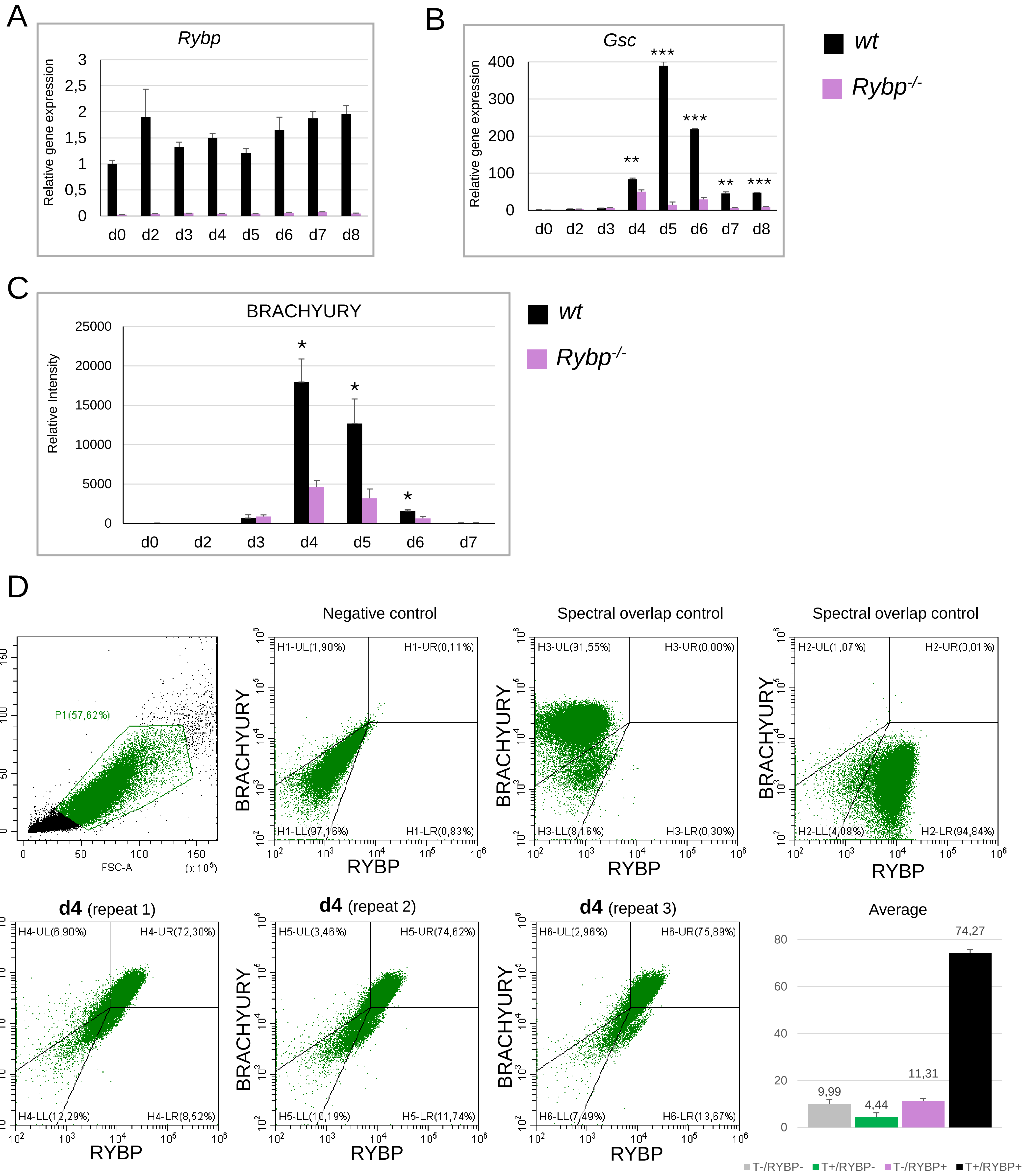

Supplementary Figure 2. The major germ layer formation is disrupted in the *Rybp*$^{-/-}$ cultures

Gene expression changes of **(A)** *Rybp* and **(B)** *Goosecloid* (*Gsc*) in *wt* and *Rybp*$^{-/-}$ cells during progenitor formation. **(C)** Relative BRACHYURY signal intensities were measured troughouth cardiac progenitor formation of *wt* and *Rybp*$^{-/-}$ samples. The intensities were counted from 3 independent experiments and normalized for corresponding DAPI stanings using Image J software. Error bars represent standard deviation, n=3, Values of p<0.05 were accepted as significant (*p<0.05; **p<0.01; ***p<0.001), Statistical method: t test type 3. **(D)** Flow cytometry plots showed 74.27% of RYBP+/GATA4+ cells in d4 cardiac differentiated samples. The average cell number was calculated from 3 biological samples.

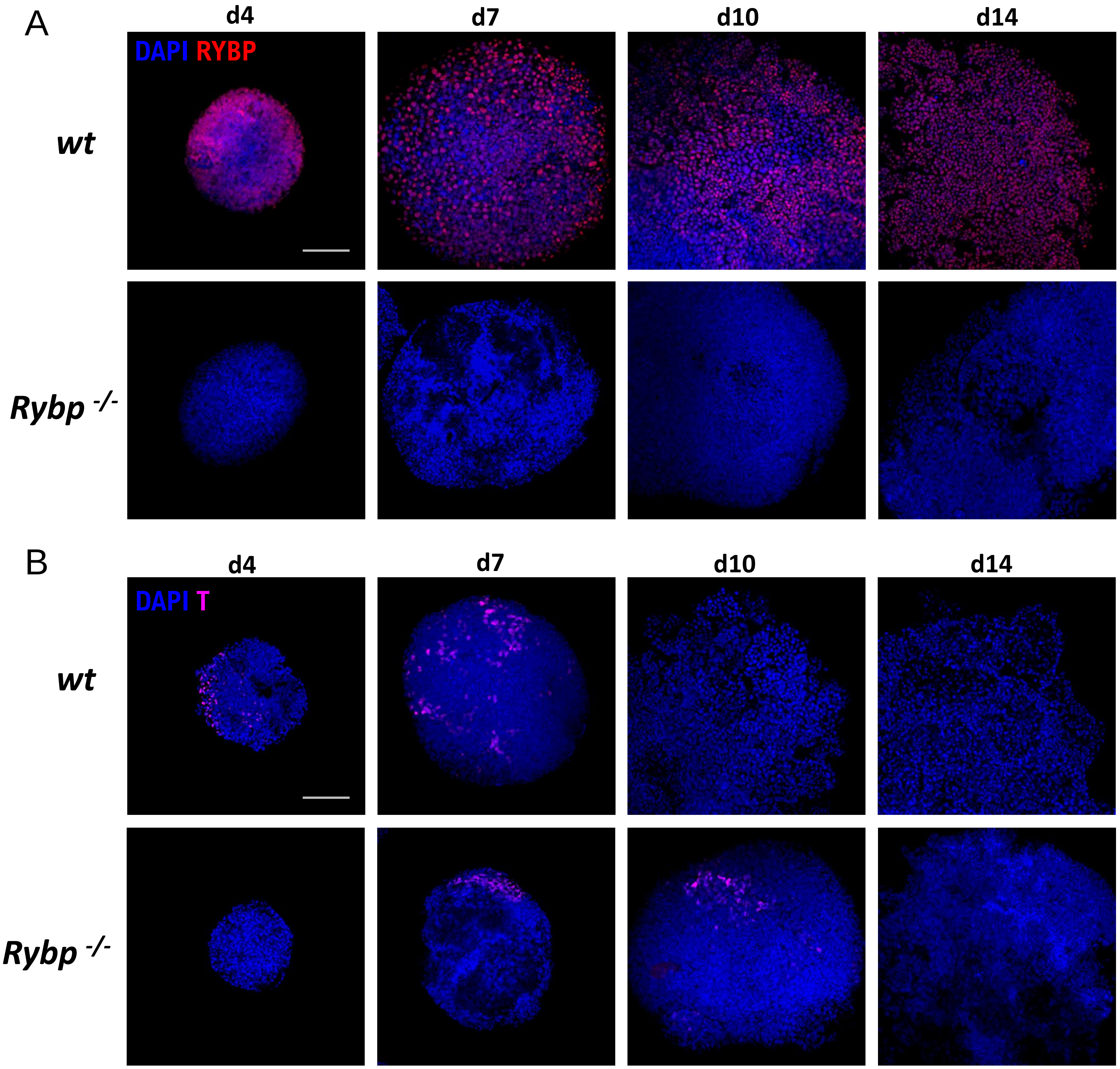

Supplementary Figure 3. DAPI counterstainings of *wt* and *Rybp*$^{-/-}$ EBs

Wt and *Rybp*$^{-/-}$ embryoid bodies were stained with DAPI for nuclear visualization. DAPI was overlapped with **(A)** RYBP, **(B)** BRACHYURY (T) Blue: DAPI, red: RYBP, magenta: T. Confocal images were taken from the surface and middle of the EBs with Olympus Confocal IX 81, Obj.: 20 x; Scale bar: 100 µm.

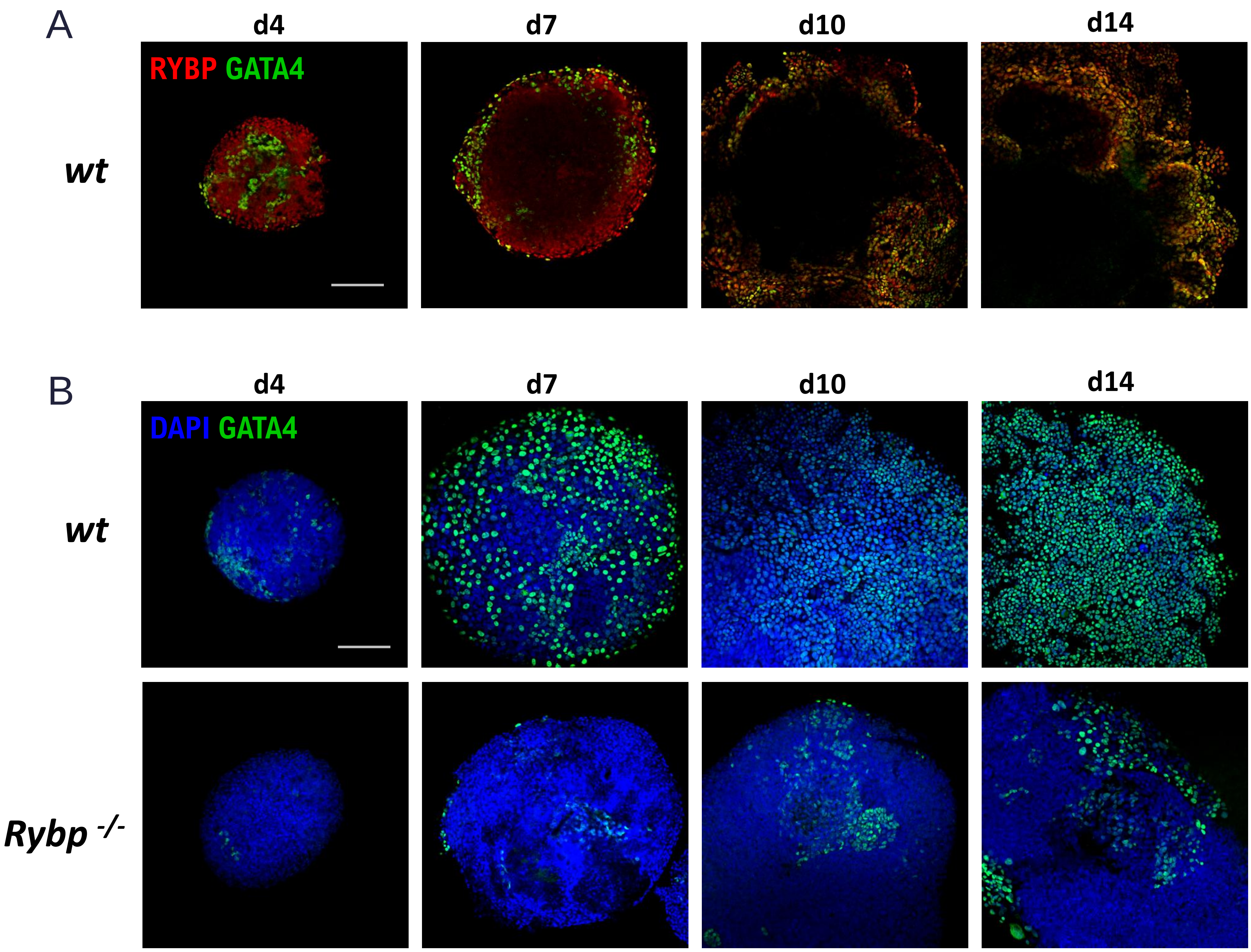

Supplementary Figure 4. Immuncytochemistry showing RYBP and GATA4 co-localization in outher leyers of *wt* EBs and DAPI counterstainings of *wt* and *Rybp*$^{-/-}$ EBs.

(A) RYBP and GATA4 co-localize in the outer leyers of *wt* EBs. **(B)** *Wt* and *Rybp*$^{-/-}$ embryoid bodies were stained with DAPI for nuclear visualization. DAPI was overlapped with GATA4. Blue: DAPI, red: RYBP, green: GATA4. Confocal images were taken from the surface and middle of the EBs with Olympus Confocal IX 81, Obj.: 20 x; Scale bar: 100 µm.

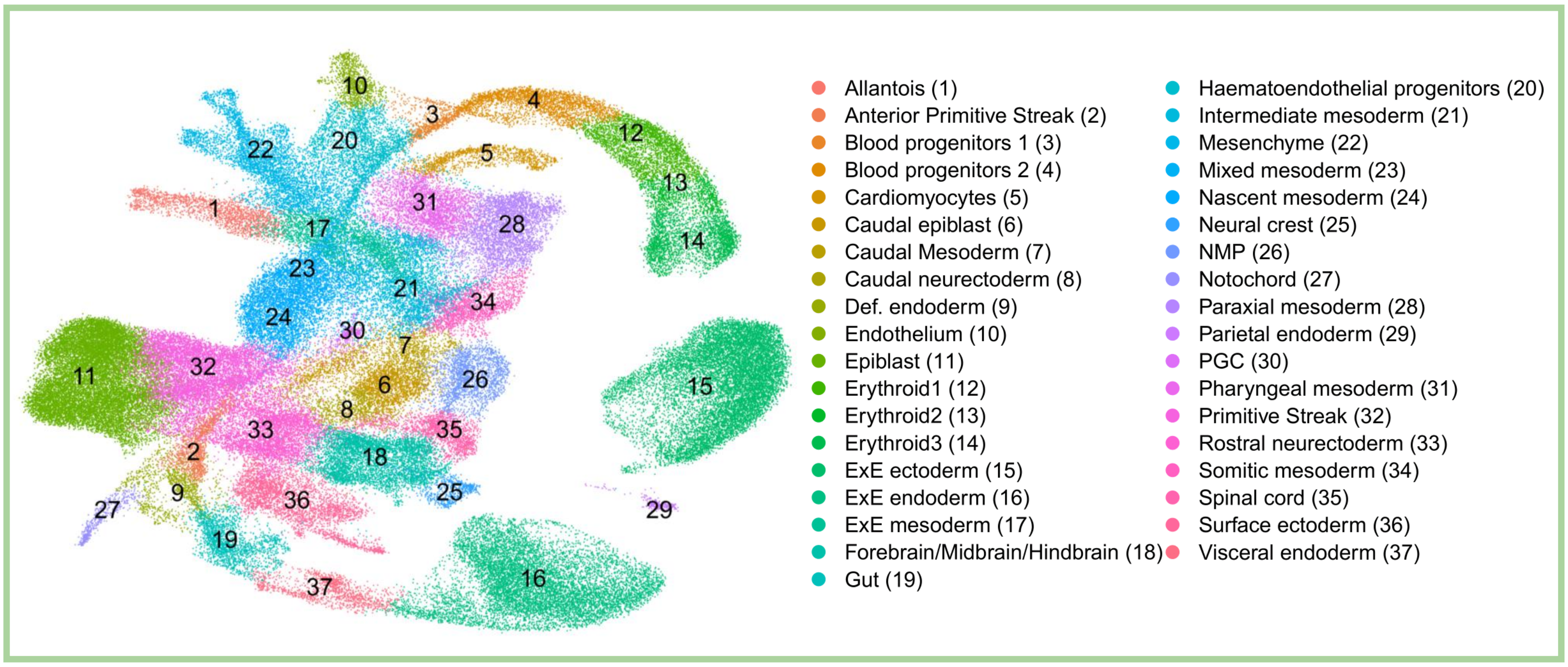

Supplementary Figure 5. UMAP plot of different cell types during gastrulation and early organogenesis

Single-cell RNA datasets were reanalyzed using a same method as described in Pijuan-Sala et al., 2019 and received the same 37 clusters, as expected.

Abbreviations: ExE: extra-embryonic, NMP: neuromesodermal progenitors, PGC: primordial germ cells, Def.: definitive.

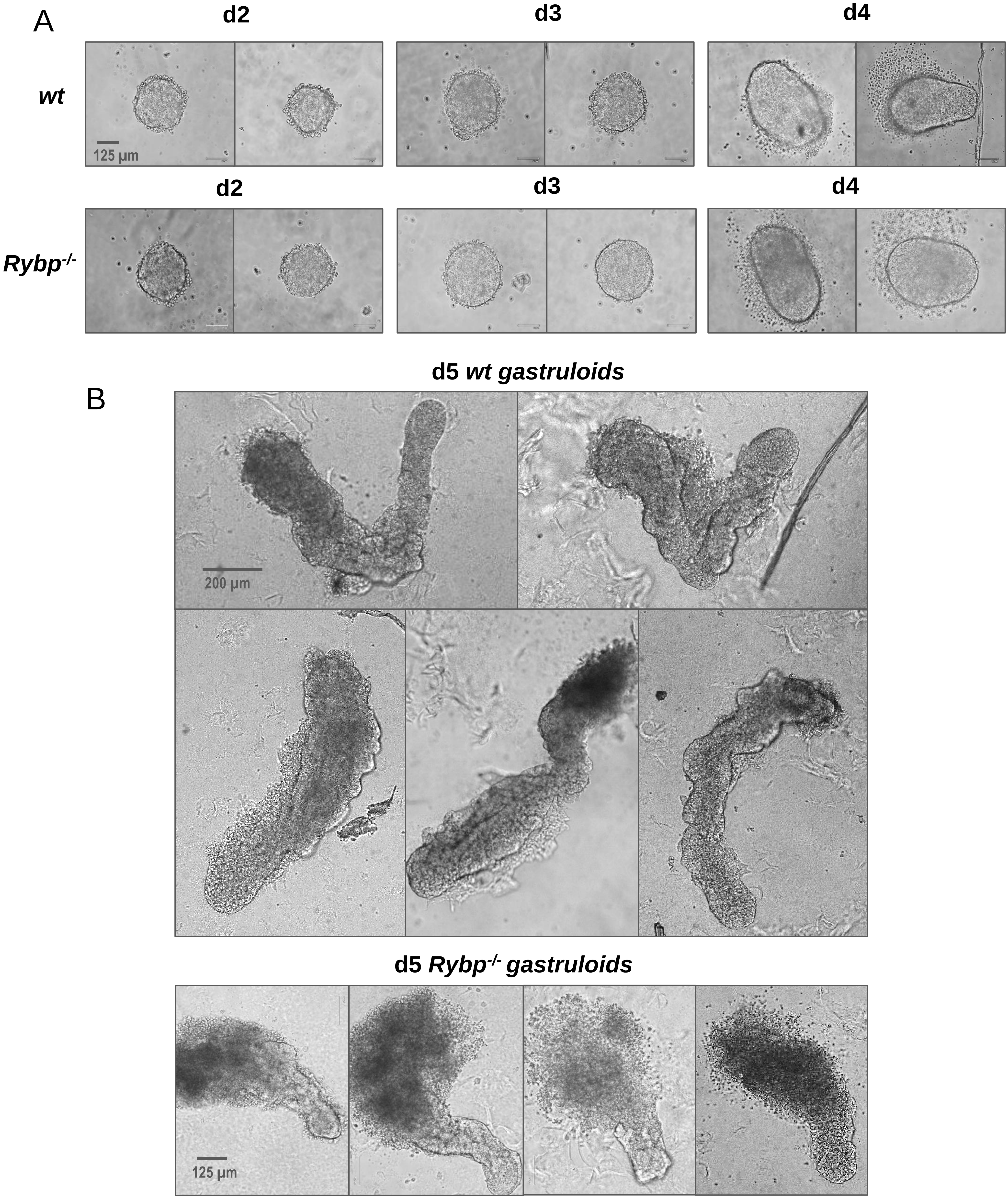

Supplementary Figure 6. ***Wt*** **and** ***Rybp***$^{-/-}$ **gastruloids exhibited different morphology**

Bright-field images of *wt* and *Rybp*$^{-/-}$ gastruloids were taken during **(A)** early (d3, d4) and **(B)** late (d5) gastruloid differentiation. Spinning Disc Confocal, Obj.: 10x, 20x, Scale bars: 125 µm, 200 µm

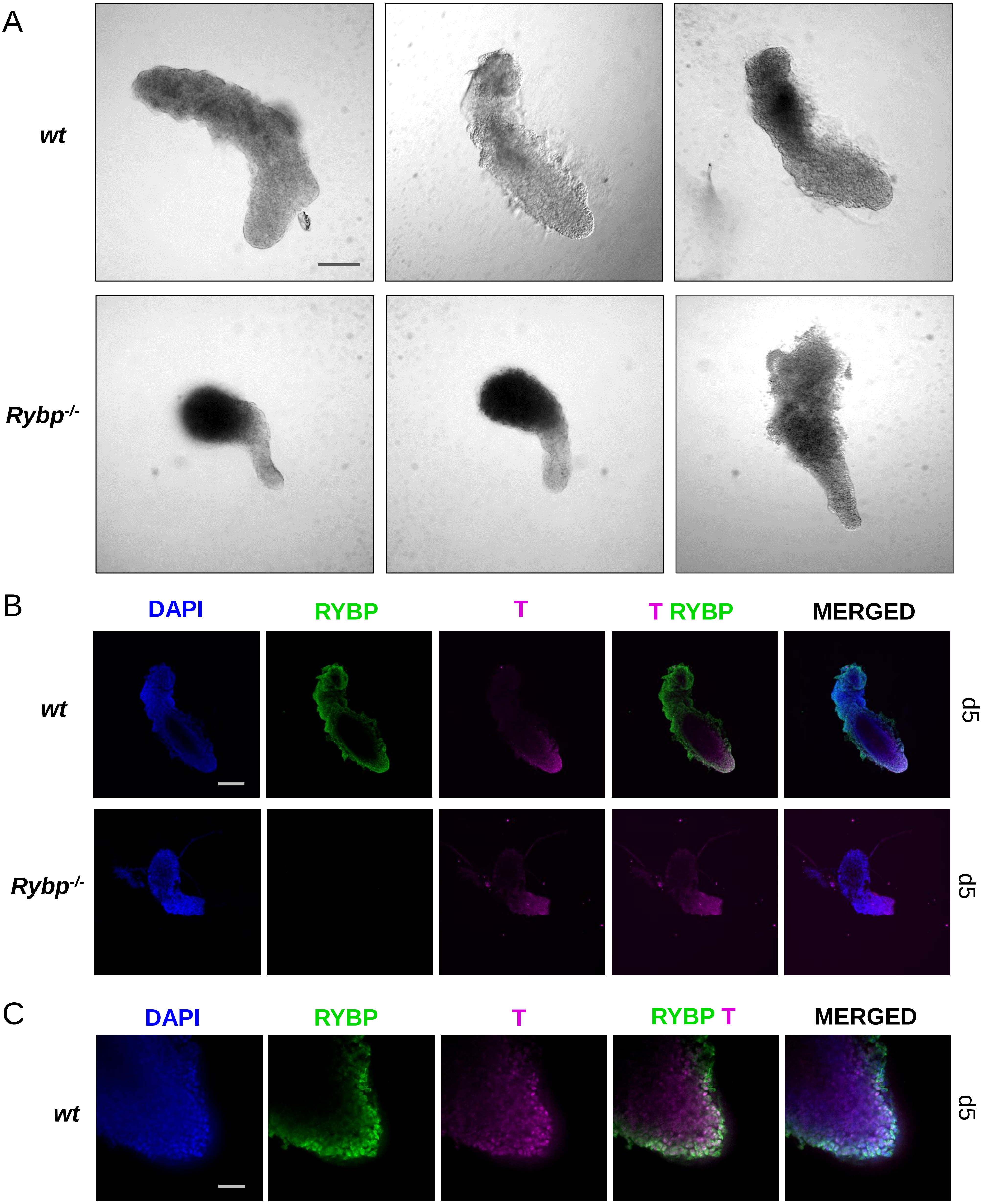

Supplementary Figure 7. Second biological samples of *wt* and *Rybp*$^{-/-}$ gastruloids

(A) Bright field images of *wt* and *Rybp*$^{-/-}$ d5 gastruloids derived from second biological sample. **(B)** d5 gastruloids were immunostained for RYBP and BRACHYURY. **(C)** RYBP and BRACHYURY showed co-localization in the most caudal region of the tailbud. Blue: DAPI, green: RYBP, magenta: T. Spinning Disc Confocal, Obj.: 10x (A-B) and 40x (C); scale bars: 200 µm (A-B), 50 µm (C)

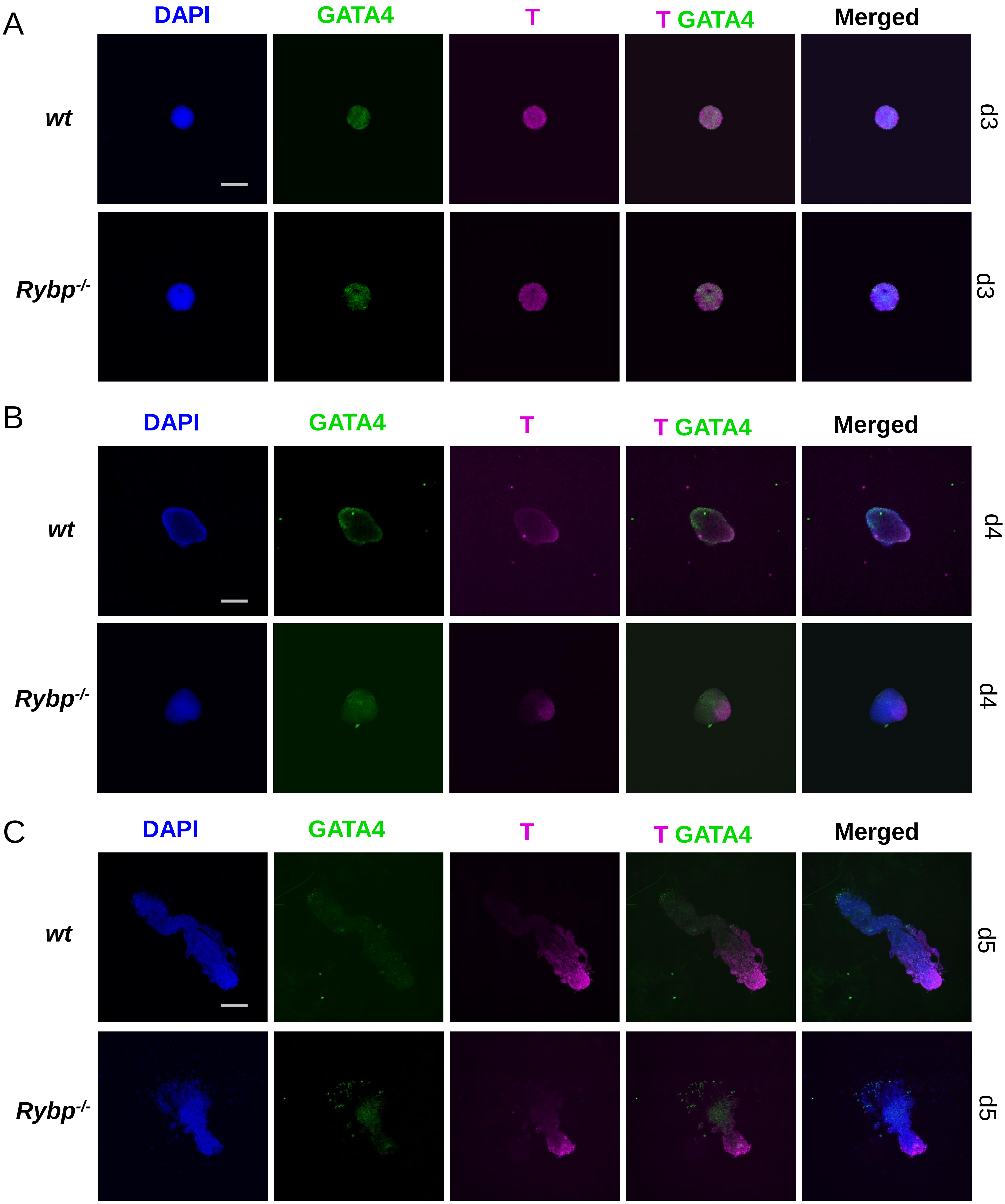

Supplementary Figure 8. The number of GATA4+ cells was decreased in *wt* and *Rybp$^{-/-}$* gastruloids

GATA4 and BRACHYURY immunocytochemistry showed **(A)** similar GATA4 protein levels in d3 *wt* and *Rybp$^{-/-}$* gastruloids. **(B)** The number of GATA4+ cells started to decrease at d4 and BRACHYURY and GATA4 were detected in opposite poles of the gastruloids. **(C)** By d5, only few GATA4+ cells were detected in the anterior region of the *wt* and *Rybp$^{-/-}$* gastruloids. Blue: DAPI, green: RYBP, magenta: BRACHYURY (T). Spinning Disc Confocal, Obj.: 10x scale bars: 50 μm

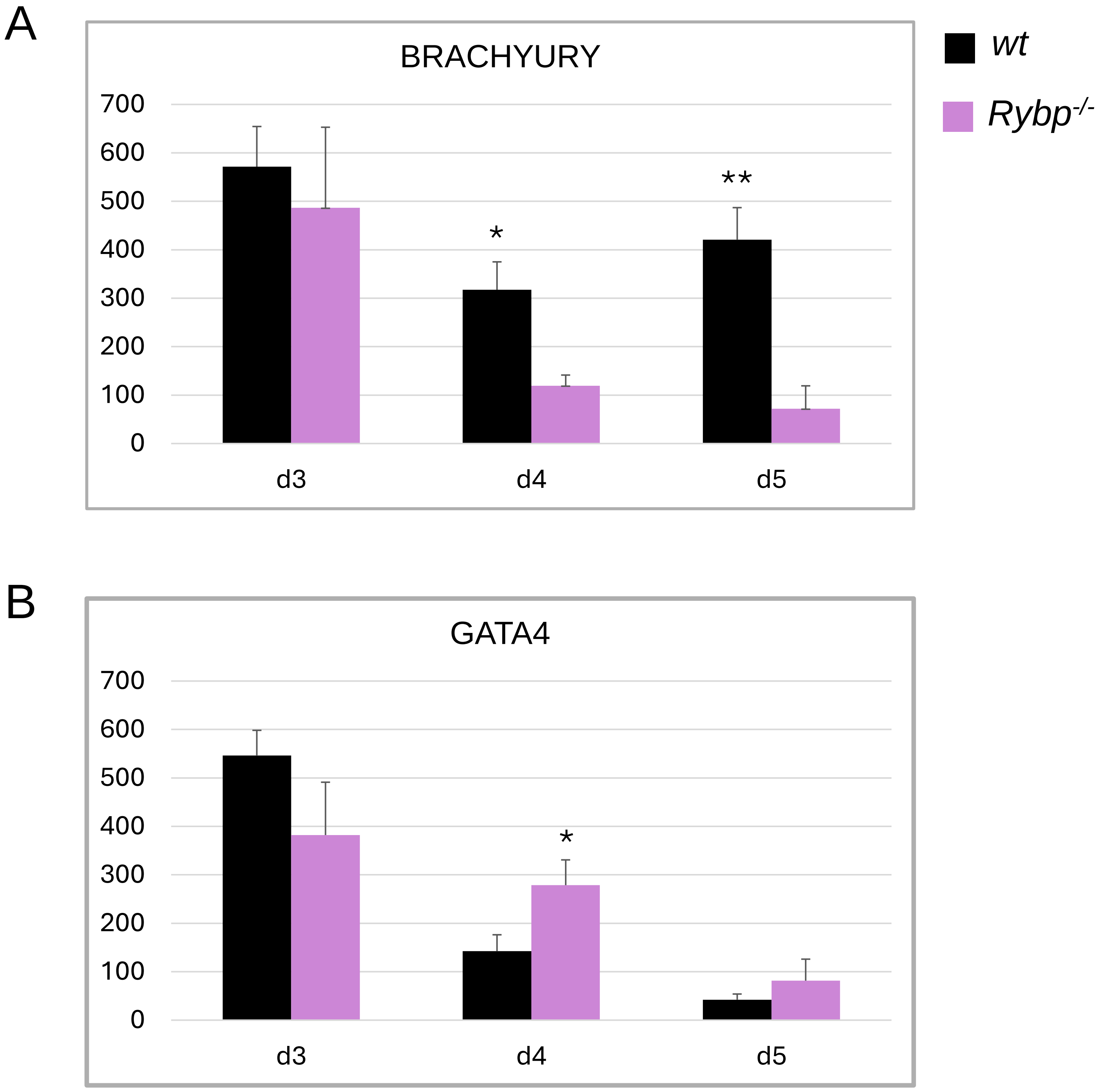

Supplementary Figure 9. Quantification of BRACHYURY and GATA4 ICC signal intensities in *wt* and *Rybp$^{-/-}$* gastruloids

(A) BRACHYURY and **(B)** GATA4 immunocytochemistry signal intensities were counted from three independent samples using Image J software. The intensity values were normalized to DAPI signal and compared to *wt* ES cells. Error bars represent standard deviation, n=3, Values of p<0.05 were accepted as significant (*p<0.05; **p<0.01; ***p<0.001), Statistical method: t test type 3.
